# Dichlorinated and Brominated Rugulovasines, Ergot Alkaloids Produced by *Talaromyces wortmannii*

**DOI:** 10.3390/molecules200917627

**Published:** 2015-09-23

**Authors:** Lívia Soman de Medeiros, José Vinícius da Silva, Lucas Magalhães Abreu, Ludwig Heinrich Pfenning, Carolina Lúcia Silva, Sérgio Secherrer Thomasi, Tiago Venâncio, Karl-Heinz van Pée, Kristian Fog Nielsen, Edson Rodrigues-Filho

**Affiliations:** 1 Department of Systems Biology, Technical University of Denmark, Søltofts Plads, Building 221, Kgs. Lyngby DK-2800, Denmark; E-Mails: lmabreu@gmail.com (L.M.A.); kfn@bio.dtu.dk (K.F.N.); 2 Department of Chemistry, Federal University of São Carlos, Rod. Washington Luís, Km 265, São Carlos 13565-905, Brazil; E-Mails: zeh.vinicius@gmail.com (J.V.S.); carolina.l@hotmail.com.br (C.L.S.); secherrer@yahoo.com.br (S.S.T.); venancio@ufscar.br (T.V.); edinho@pq.cnpq.br (E.R.-F.); 3 Department of Phytopathology, Federal University of Lavras, P. O. Box 3037, Lavras 37200-000, Brazil; E-Mail: ludwig@ufla.br; 4 Allgemeine Biochemie, Technical University of Dresden, Bergstraβe 66, Dresden 01062, Germany; E-Mail: Karl-Heinz.vanPee@chemie.tu-dresden.de

**Keywords:** halogenation, tryptophan, rugulovasine, dereplication, *Talaromyces*

## Abstract

UHPLC-DAD-HRMS based dereplication guided the detection of new halogenated alkaloids co-produced by *Talaromyces wortmannii*. From the fungal growth in large scale, the epimers 2,8-dichlororugulovasines A and B were purified and further identified by means of a HPLC-SPE/NMR hyphenated system. Brominated rugulovasines were also detected when the microbial incubation medium was supplemented with bromine sources. Studies from 1D/2D NMR and HRMS spectroscopy data allowed the structural elucidation of the dichlorinated compounds, while tandem MS/HRMS data analysis supported the rationalization of brominated congeners. Preliminary genetic studies revealed evidence that FADH_2_ dependent halogenase can be involved in the biosynthesis of the produced halocompounds.

## 1. Introduction

Natural compounds containing carbon–halogen bonds have been discovered for years but were often considered rare or isolated as artifacts in the past [1,2]. However, many halogenase enzymes from microorganisms have been identified and characterized. Presently, it is estimated that around 5000 natural halometabolites have been discovered, with the alkaloids responsible for approximately 25% of these [1,3]. Since nitrogen-containing chemical structures are excellent platforms for biohalogenation, the discovery of new halogenated alkaloids is expected [3]. Their production is particularly highlighted in the marine environments due to the higher availability of the chloride and bromide species [4,5], even though terrestrial organisms including some fungi species were also described as organohalogens producers [6].

**Figure 1:**
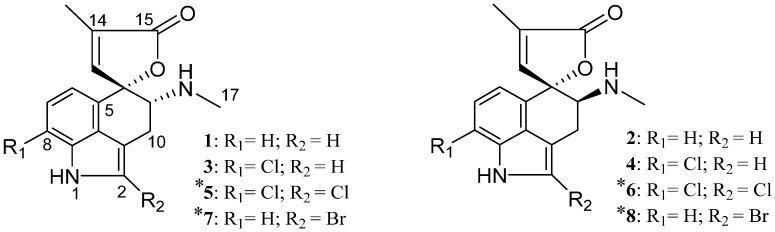
Chemical structures of the alkaloids produced by the *T. wortmannii*: (**1**) rugulovasine A; (**2**) rugulovasine B, (**3**) 8-chlororugulovasine A; (**4**) 8-chlororugulovasine B; (**5**) 2,8-dichlororugulovasine A; (**6**) 2,8-dichlororugulovasine B; (**7**) 2-bromorugulovasine A; and (**8**) 2-bromorugulovasine B. * Alkaloids reported for the first time. The depicted structures represent only relative configuration.

The usage of exploratory strategies has increased notably within natural products research, including for instance, the ultrahigh performance liquid chromatography-diode array detection—high resolution mass spectrometry (UHPLC-DAD-HRMS) and tandem high resolution mass spectrometry (MS-HRMS) based dereplication [7,8]. Such approaches usually comprise dedicated data evaluation [9,10] but often result in successful and rapid identification of known compounds, revealing secondary metabolites still untapped [11,12,13]. In view of the foregoing outcomes, the metabolite screening of *Talaromyces wortmannii* (*T. wortmannii*) extracts was performed trough ultraviolet/visible (UV/Vis) and HRMS data analysis in combination with queries in commercial and in-house microbial databases. The strategy led to the identification of tryptophan-derived alkaloids, comprised of new halogenated rugulovasines (Figure 1). Rugulovasines are modified clavine alkaloids containing a singular spirolactone moiety [14], with toxic effects observed in day-old chickens [15,16]. These metabolites have been isolated as a racemic mixture [14,17] since they may interconvert upon warming through vinylogous Mannich reaction. Due to this chemical feature, they have also attracted interest in organic synthesis studies [18,19]. The first occurrence of a halocompound derived from rugulovasines was reported by Cole in the 1970s [17] and so far, the new halogenated alkaloids produced by *T. wortmannii* are unique representatives to be reported since Cole’s discovery.

Usually, the numbering of carbons into the ergot alkaloid backbone is established according to the original ergoline system [20]. Nevertheless, in their first report, the 8-chlororugulovasines were named from a different counting system [17], where the new halogenated rugulovasines were named on the basis of their known analogues.

## 2. Results and Discussion

The fungus *T. wortmannii* (CML 2704) was isolated from symptomless apple fruits (*Malus domestica*), following standard procedures for isolation of endophytic fungi [21]. Different growth media were tested for the incubation of the microorganism: PDA, CREA, MEA, CYA, DRYES, MEAox, WATM, YES and OA (for definitions see Section 3.5). The obtained micro-extracts had their metabolite screening carried out by UHPLC-DAD-QTOFMS (quadrupole time-of-flight mass detector) data analysis. A thorough investigation was applied from the observed accurate monoisotopic masses, fragment ions and adduct patterns, which assisted the finding of the elemental composition from the detected peaks in the base peak chromatograms (BPC). The found molecular formulas and accurate masses (±5 ppm) in the database AntiBase 2012 [22] as well as the use of a comprehensive microbial standard collection [9,10] were concomitantly applied along the data analysis process. Owing to the similar chemical profile exhibited in the chromatograms from the investigated micro-extracts, the PDA medium was chosen as the working growth medium and likewise for the results discussed hereafter.

**Figure 2:**
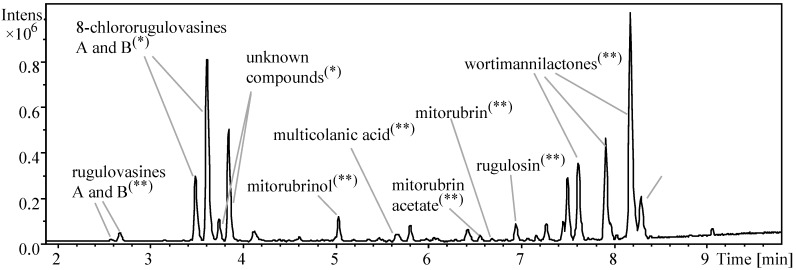
BPC chromatogram (100–1000 Da) from the micro-extract of *T. wortmannii* growth in PDA medium. Compounds identified by UV/Vis-HRMS based dereplication are indicated. (*) Isolated compounds. (**) compounds confirmed by comparison with reference standards. Peaks not highlighted have not been unambiguously identified. Data acquired at electrospray ionization in positive mode (ESI+), UHPLC-DAD-QTOFMS instrument.

Among the hits retrieved by the database, the presence of multicolanic acid, (+)-mitorubrin, (−)-mitorubrinol, (+)-mitorubrinol acetate, skyrin, rugulosin, and rugulovasine A and B, was confirmed in the chromatograms by the comparison with the authentic reference standards. The overview from the confirmed AntiBase hits produced by *T. wortmannii* in PDA medium is shown in Figure 2. Still including the main detected peaks, the 8-chlororugulovasines A and B were also found as likely records in the database. Since those compounds were not available in the standard collection, they were isolated from the scaled-up cultivation of the strain and confirmed on the basis of spectroscopic analysis, as will be further described. Apart from the identified compounds, two peaks eluted around 3.7 to 3.9 min in the BPC chromatogram (Figure 2), did not provide potential candidates, either in AntiBase or in the further literature queries. These observations led to the hypothesis of compounds biosynthesized by *T. wortmannii* still to be elucidated.

### 2.1. Known and New Halogenated Rugulovasines Produced by T. wortmannii

**Figure 3:**
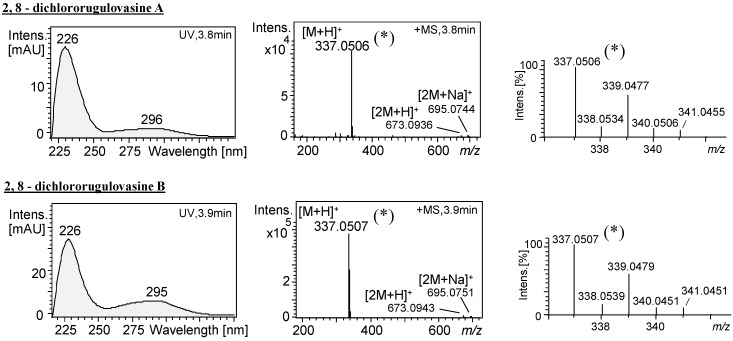
UV/Vis and *full scan* spectra of 2,8-dichlororugulovasine A and B produced by *T. wortmannii* in PDA incubation medium. (*) Magnified signal from pseudomolecular ion [M + H]^+^ and corresponding isotopes. Data acquired at ESI+, UHPLC-DAD-QTOFMS instrument.

Besides the production of rugulovasine A and B by *T. wortmannii*, the dereplication process pointed towards the co-production of the halocongeners 8-chlororugulovasines A and B. This evidence was provided by the detection of the accurate masses *m*/*z* 303.0892 (−1.0 ppm) and 303.0896 (−0.3 ppm) (Figure S1), where the elementary compositions were given by the same formula C_16_H_16_O_2_N_2_Cl. The occurrence of halogenated compounds in the fungal micro-extracts was suggested not only by the likely detection of the known monochlorinated alkaloids but also due to the isotope pattern displayed by the two unknown ions detected at 3.7 and 3.9 min (Figure 3). Their accurate masses *m*/*z* 337.0506 (−0.2 ppm) and 337.0507 (−0.6 ppm) afforded the molecular formula C_16_H_15_O_2_N_2_Cl_2_ for both, which, notably, matched to two additional chlorine atoms when compared to rugulovasines chemical composition (C_16_H_17_O_2_N_2_). Indeed, in accordance with the theoretical isotopic pattern found for the C_16_H_15_O_2_N_2_Cl_2_, the observed ions fitted to dichlorinated species.

Moreover, similarly to the UV/Vis spectra from the rugulovasines and chlororugulovasines, the new compounds also showed a UV spectrum profile typical to tryptophan derivatives, with absorption maximums at nearly 225 and 295 nm (Figure S1). Altogether, these features indicated new alkaloids potentially produced by the microorganism that were later identified as rugulovasine congeners, 2,8-dichlororugulovasine A and 2,8-dichlororugulovasine B.

Rugulovasines A and B as well as 8-chlororugulovasines A and B are reported as interconverting racemic forms favored by vinylogous Mannich type reaction [14,17,19]. Based on NMR and crystallography measurements, Cole and coworkers [17] established the absolute configuration of the rugulovasines series and further showed that in normal phase chromatography, stereoisomers A are more polar than stereoisomers B, likely due to the different conformations assumed by each structure. In other words, the antiperiplanar arrangement that the isomers A undergo, which is not achieved by the isomers B [18], can lead to particular physicochemical interactions with the stationary phase and likely cause earlier elution of the diasteroisomer A prior to B in reversed-phase chromatography. Based on this, we propose that the new dichlorinated rugulovasines A and B follow the same pattern and we thus assign the relative configuration of the first peak to A and the second to B, according to their retention in the reversed phase chromatographic systems used in our work.

In order to isolate the 8-chlororugulovasines A and B and the new metabolites to confirm their chemical structures based on NMR and HRMS data analysis, the fungal strain was cultivated in large scale. The scaled-up extract was submitted to medium pressure chromatography, giving rise to enriched fractions in the target compounds. The final purification of the target alkaloids showed to be a laborious task due to the low yield of these metabolites when compared to the polyketides co-produced by the strain, and in particular the occurrence of signal broadening favored by the interconvertion of the isomers A and B. To overcome this, the most enriched fraction in the halometabolites was submitted to an automated coupled HPLC-SPE system with the sequential analysis by NMR with a cryogenic probehead. Along the chromatographic run, the compounds were trapped in polydivinylbenzene SPE cartridges, in a total of 30 sequential injections.

Afterwards, the isolated compounds were then eluted from the SPE cartridges with methanol-*d*_4_ before 1D and 2D NMR spectroscopy analysis. The NMR spectroscopic data allowed the identification of the known 8-chlororugulovasines A and B and the structural elucidation of the 2,8-dichlororugulovasine A and B (data are fully depicted in Figures S2–S31). The data set from the new dichlorinated alkaloids is summarized in Table 1, whereas from the known monochlorinated species are detailed in the Supplementary Material, Table S1.

**Figure 4:**
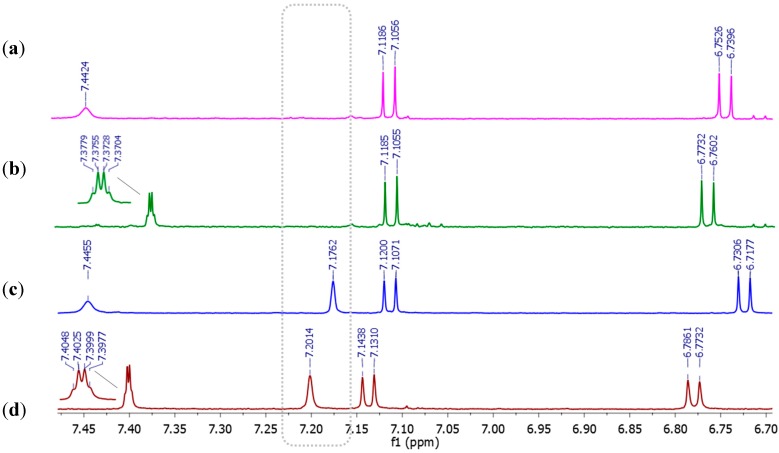
Amplified regions from the ^1^H-NMR spectra of (600 MHz, methanol-*d*_4_) (**a**) 2,8-dichlororugulovasine B; (**b**) 2,8-dichlororugulovasine A; (**c**) 8-chlororugulovasine B; and (**d**) 8-chlororugulovasine A. Region of vinylic protons is highlighted.

As expected, the ^1^H-NMR data analysis revealed a very similar chemical profile among the isolated compounds. In summary, the signals presented typical chemical shifts belonged to aromatic, vinylic, methylene and methyl systems, as well as NH indol nucleus. Although these sort of signals were similarly encountered for all metabolites, the absence of one singlet into the interval of δ_H_ = 7.17 and 7.20 ppm was noteworthy when the ^1^H-NMR spectra from the new compounds was compared to the monohalogenated ones. In other words, by the overview from the magnified regions related to the deshielded nuclei (Figure 4), broad singlets were clearly observed for the 8-chlororugulovasines while they were not detected for the 2,8-dichlororuguvasines. These singlets were assigned to the vinyl protons belonged to the indole system from the monochlorinated analogues and hence this was the first evidence that pointed out the likely site for the second chlorine unit regarding the new structures.

The key information was nonetheless given by the nuclei disposal at their aromatic system and by the couplings found for the remaining vinyl and methyl protons present in the unknown compounds backbone. The ^1^H spectra from the new metabolites (Figure 4a,b) exhibited the presence of two ortho-positioned aromatic protons assigned by H-6 and H-7. Regarding the isomer B, they were represented by the pair of dublets at δ_H_ = 7.11 ppm and 6.74 ppm with coupling constant of *J* = 7.8 Hz, while the dublets δ_H_ = 7.11 ppm and 6.77 ppm with *J* = 7.8 Hz were clearly seen in ^1^H-NMR spectrum belonged to the epimer A. This result comprised not only the hydrogens placement in the aromatic ring system of the new metabolites, but also assisted with the confirmation of one chlorine atom attached to their C-8 carbon nucleus, since the data from the 8-chlororugulovasines showed the same set of signals.

**Table 1:** ^1^H-NMR and HMBC data (600 MHz, methanol-*d*_4_) of 2,8-dichlororugulovasine A and B.

Position	2,8-Dichlororugulovasine A			2,8-Dichlororugulovasine B		
	δ_H_ (*J* in Hz)	δ_C_	HMBC	δ_H_ (*J* in Hz)	δ_C_ *	HMBC *
1	8.09 (s)	-	-	8.09 (s)	-	-
2	-	121.2 *	-	-	120.8	-
3	-	n.d.	-	-	106.3	-
4	-	129.2 *	-	-	128.3	-
5	-	125.9 *	-	-	125.0	-
6	6.77 (d, 7.8)	117.7	C-4 *, C-8 *	6.74 (d, 7.8)	115.6	C-4,C-8, C-11
7	7.11 (d, 7.8)	123.6	C-5 *, C-8 *, C-9	7.11 (d, 7.8)	122.0	C-5,C-8, C-9
8	-	117.7 *	-	-	116.2	-
9	-	131.8 *	-	-	130.8	-
10a	3.21–3.28 (n.i.) *	23.2 *	C-2 *, C-4 *, C-11 *	3.15–3.23 (n.i.) *	23.1	C-2, C-3, C-4
			C-12*			C-12
10b	2.89–2.92 (m) *	23.2 *	-	2.90–2.97 (m) *	23.1	C-3, C-11
11	3.21–3.28 (n.i.) *	63.4	C-12 *	3.15–3.23 (n.i.) *	63.05	C-12
12	-	88.5	-	-	88.7	-
13	7.37 (br q, 1.4)	151.8	-	7.44 (br s)	150.4	-
14	-	131.6	-	-	130.5	-
15	-	175.8	-	-	174.1	-
16	2.02 (d, 1.5)	11.4	C-13, C-14, C-15	1.98 (d, 1.5)	8.97	C-13, C-14, C-15
17	2.44 (s)	35.6	C-11	2.42 (s)	33.60	C-11

^13^C shifts obtained by the signal projection in the heteronuclear single quantum correlation (HSQC) and in the heteronuclear multiple quantum correlation (HMBC) spectra; * Signals and correlations obtained in the ^1^H-NMR and HMBC spectra from 2,8-dichlororugulovasine A and B mixture; n.d.: signal not detected; n.i.: multiplicity not identified due to overlapping of solvent signal.

Furthermore, the quadruplet observed at δ_H_ = 7.37 ppm (H-13) for the epimer A and the broad singlet at δ_H_ = 7.44 ppm (H-13) for epimer B allowed the proposal of an allylic group as part of the spiro system. The observed multiplicities suggested the coupling between the vinylic and methyl nuclei, while the correlations found in the HMBC spectrum from 2,8-dichlororugulovasine A indicated the attachment of these protons to the spiro ring. For instance, it was detected the correlation of CH_3_-16 (δ_H_ = 2.02 ppm) to the nuclei C-13 (δ_C_ = 151.8 ppm), as well as to C-14 (δ_C_ = 131.6 ppm) and C-15 (δ_C_ = 175.8 ppm).

Another piece of information provided by HMBC data, showed the long-correlation of H-2 to the carbon C-9 from the indole scaffold of 8-chlororugulovasine A, whereas such nuclei interaction was not observed regarding the compound 2,8-dichlororugulovasine A (Figure S32). As a whole, the previous signal assignments precluded hereby the aromatic ring and the spiro system as the moieties for the binding of the second expected chlorine atom. Therefore, the indol nucleus C-2 represented the remaining sp^2^ carbon most suitable for the halogenation. The same backbone disposal was confirmed to the isomer B, based on the same chemical profile obtained in ^1^H-NMR data (Table 1) and also the MS/HRMS data analysis showed hereafter. The signal assignments of isolated structures showed to be in agreement to the data previously reported for the rugulovasine congeners [15,17].

### 2.2. Brominated Rugulovasines and MS/HRMS Data from the Co-Produced Rugulovasines

Halogen atoms can be incorporated into organic compounds by enzyme-catalyzed reactions with halide ions [23]. However, the halogen binding is usually determined by the relative amount of halide present in the environment where the producer is submitted. For instance, the bromometabolites are most commonly biosynthesized by marine organisms, while chlorometabolites are predominately produced by terrestrial organisms [23,24]. Despite the fact that *T. wortmannii* has been isolated from apple fruits, the use of brominated additives into the potato dextrose (PD) medium culture was tested owing to verify the likely biosynthesis of brominated compounds by the strain. As a first attempt, the acid HBr was used alternatively to HCl for pH correction of culture media (final pH = 5.6). Separately, the second experiment employed the incorporation of KBr into the fermentation medium (2 mg/L), with no buffer. The obtained samples were then preliminarily analyzed via MS direct infusion (low-resolution data).

**Figure 5:**
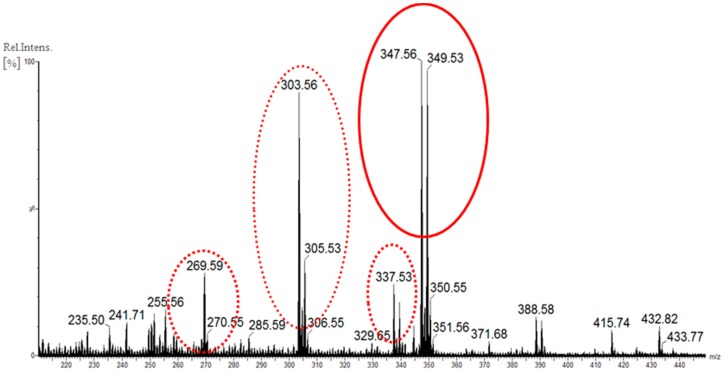
Full scan spectrum of the crude extract obtained from *T. wortmannii* cultivated in PD medium with HBr as additive. Pseudomolecular ions from the co-produced rugulovasines and chlorinated analogues are highlighted in dashed line, while the brominated detected species is highlighted in continuous line. Data acquired at ESI+, triple quadrupole MS instrument.

Regarding the HBr addition into the incubation medium, the co-production of bromometabolites with rugulovasines congeners was readily apparent in the full scan spectrum of the corresponding extract (Figure 5). The diagnostic ions for the target alkaloids *m*/*z* 269, 303 and 337 were concomitantly detected to the ions *m*/*z* 347 which displayed the typical isotope pattern of a monobrominated compound. Likewise, the same outcome was encountered for the experiment using KBr into the medium instead of HBr, as illustrated in Figure S33. The found nominal mass *m*/*z* 347 matched to 44 Da and 78 Da mass differences from the 8-chlororugulovasines and rugulovasines pseudomolecular ions, respectively, indicating the attachment of one bromine unit rather than one chlorine or one hydrogen atom to the unknown metabolite. Such observation was thus supported by the inspection of the UHPLC-HRMS data, which afforded the two peaks of accurate masses *m*/*z* 347.0389 (−0.1 ppm) and *m*/*z* 347.0388 (−0.3 ppm) (Figure S34). Both ions had the molecular formula suggested by C_16_H_16_O_2_N_2_Br, which fit the acquired experimental and theoretical isotope patterns.

**Figure 6:**
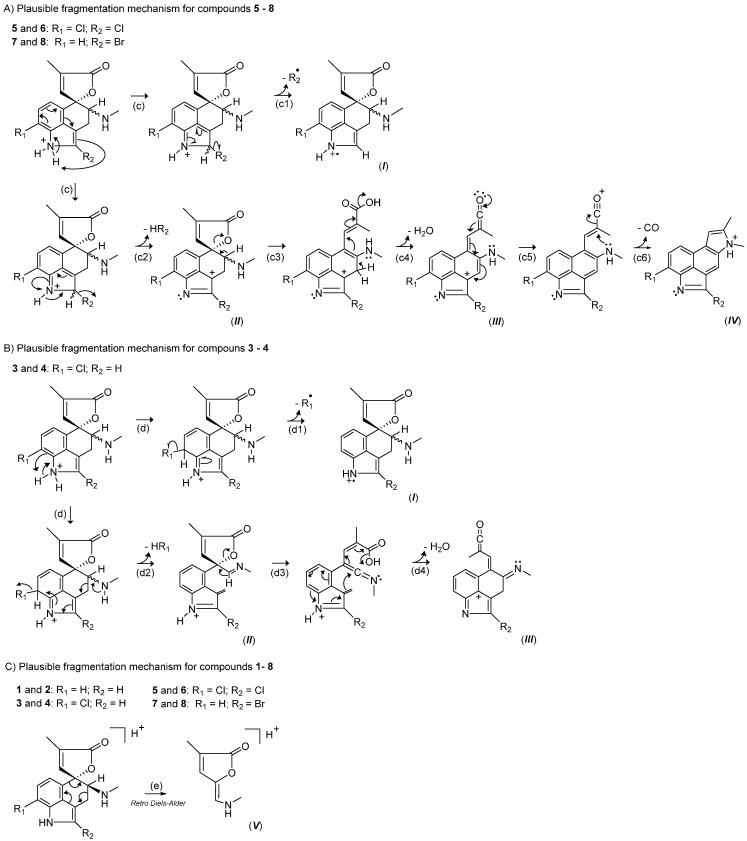
Plausible fragmentation mechanisms proposed to explain the product ions detected in the MS/HRMS spectra from (**A**) compounds **5**–**8**; (**B**) compounds **3** and **4**; and (**C**) compounds **1**–**8**.

Therefore, the fungal extract supplemented with HBr was then submitted to MS/HRMS data analysis in view of establishing their fragmentation profile and to assist the characterization of the brominated chemical structures through the comparison with the compounds previously isolated. Differently from the dereplication process, the product ions experiment was carried out using HRMS instrument equipped with an Orbitrap mass analyzer (FTMS) whose the fully spectroscopic data are found in the Supplementary Material (Figures S35–S46). The common rugulovasine backbone was then confirmed for all co-produced alkaloids altogether, on the basis of the fragmentation pattern established by the detected accurate masses in their MS/HRMS spectra. The product ions that justify the rugulovasine scaffold are summarized in Table S2, while the plausible fragmentation mechanisms are detailed in Figure S49. Additionally, regarding the chlorinated and brominated species, the MS/HRMS data analysis also enabled the characterization of the halogenation sites into the tryptophan core and in particular the rationalization of the 2-bromorugulovasines structures. The product ions originated from the halogen losses are listed in Table 2 and are supported by proposed fragmentation mechanisms illustrated in Figure 6.

From the pseudomolecular ions [M + H]^+^, the leaving of chlorine or bromine as radical species give rise to the product ions represented by ***I*** (Figure 6A, steps c_1_ and d_1_), while the exit of the halide group establish the ions represented by ***II*** (Figure 6A, steps c_2_ and d_2_). These losses lead to re-establishment of the aromatic system and are detected independently of the halocompound, although their occurrence might be justified trough distinct mechanisms. For example, to the 2,8-dichlororugulovasines and the 2-bromorugulovasines pseudomolecular ions, it is assumed the leaving of the halogen connected to the nucleus C-2. This pathway comprises the tertiary carbocation, which supports the stability of the ions described by ***II*** in both cases. On the other hand, for the 8-chlororugulovasines (Figure 6B), the electronic delocalization along the ergoline system is activated from the leaving of the proton belonged to the free amino group (step d_2_). The proposed mechanism affords the exit of the chlorine atom formerly attached to the nucleus C-8, maintaining the charge localized within the nitrogen moiety. In this case, the fragment ions indicated by ***II*** reach herein lower stability when compared to the species ***II*** generated from the halogenated structures at C-2, according to the relative intensities observed for them in Table 2.

**Table 2:** Summary of accurate masses from the compounds **1**–**8** and their product ions (first line). Relative intensities based on from the base peak ion in the MS/HRMS spectra (second line). Elemental composition obtained from of each accurate masses (third line).

Compound	[M + H]^+^	Product Ions *				
		(*I*)	(*II*)	(*III*)	(*IV*)	(*V*)
(1) rugulovasine A	269.1284	-	-	-	-	-
	(72%)	-	-	-	-	-
	C_16_H_17_O_2_N_2_	-	-	-	-	-
(2) rugulovasine B	269.1284	-	-	-	-	140.0705
	(37%)	-	-	-	-	(8%)
	C_16_H_17_O_2_N_2_	-	-	-	-	C_7_H_10_O_2_N
(3) 8-chlororugulovasine A	303.0894	268.1208	267.1127	249.1022	-	-
	(55%)	(5%)	(3%)	(8%)		
	C_16_H_16_O_2_N_2_Cl	C_16_H_16_O_2_N_2_	C_16_H_15_O_2_N_2_Cl	C_16_H_13_ON_2_	-	-
(4) 8-chlororugulovasine B	303.0894	268.1205	267.1122	249.1021	-	140.0705
	(17%)	(3%)	(3%)	(12%)	-	(17%)
	C_16_H_16_O_2_N_2_Cl	C_16_H_16_O_2_N_2_	C_16_H_15_O_2_N_2_Cl	C_16_H_13_ON_2_	-	C_7_H_10_O_2_N
(5) 2,8-dichlororugulovasine A	337.0503	302.0814	301.0736	283.0631	255.0679	-
	(73%)	(16%)	(68%)	(75%)	(5%)	-
	C_16_H_15_O_2_N_2_Cl_2_	C_16_H_15_O_2_N_2_Cl	C_16_H_14_O_2_N_2_Cl	C_16_H_12_ON_2_Cl	C_15_H_12_N_2_Cl	-
(6) 2,8-dichlororugulovasine B	337.0501	302.0812	301.0734	283.0628	255.0677	140.0703
	(35%)	(5%)	(27%)	(33%)	(6%)	(18%)
	C_16_H_15_O_2_N_2_Cl_2_	C_16_H_15_O_2_N_2_Cl	C_16_H_14_O_2_N_2_Cl	C_16_H_12_ON_2_Cl	C_15_H_12_N_2_Cl	C_7_H_10_O_2_N
(7) 2-bromorugulovasine A	347.0388	268.1205	267.1128	249.1021	221.1072	-
	(37%)	(13%)	(21%)	(11%)	(5%)	-
	C_16_H_16_O_2_N_2_Br	C_16_H_16_O_2_N_2_	C_16_H_15_O_2_N_2_	C_16_H_13_ON_2_	C_15_H_13_N_2_	-
(8) 2-bromorugulovasine A	347.0387	268.1206	267.1128	249.1021	221.1072	140.0705
	(13%)	(14%)	(31%)	(14%)	(4%)	(13%)
	C_16_H_16_O_2_N_2_Br	C_16_H_16_O_2_N_2_	C_16_H_15_O_2_N_2_	C_16_H_13_ON_2_	C_15_H_13_N_2_	C_7_H_10_O_2_N

Data acquired at ESI+, UHPLC- OrbitrapMS instrument. * Recorded at 25 eV.

Along the step c6, the loss of CO unit encloses one new indol ring, supporting the species represented by ***IV***. Those species are similarly observed to the new halometabolites but nonetheless absent in 8-chlororugulovasines MS/HRMS data. Moreover, still taking into account the data summarized in Table 2, the detection of the product ions highlighted by ***V*** seemed to distinguish the epimeric forms A from B from the indole alkaloids produced by *T. wortmannii*. The spacial conformation assumed by the isomers B enables the retro Diels–Alder mechanism (Figure 6C), giving rise to the diagnostic fragment ion of *m*/*z* 141 (nominal mass).

### 2.3. Detection of the Halogenase Gene

Enzymatic halogenation via hypohalite can be catalyzed using molecular oxygen as the oxidant with flavin as the redox active cofactor, in an enzimatic setting known as flavin adenine dinucleotide (FADH_2_) dependent halogenases [24,25]. It is known that FADH_2_ dependent halogenases expressed in prokaryotes are responsible for halogenation at C-5, C-6, C-7 and C-8 positions at indol moieties [26]. In contrast to prokaryotic systems, the halogenation of tryptophan is an unusual reaction in fungi. Even though the FADH_2_ dependent halogenases have been described in eukaryotic organisms, with high similarity to those found in bacteria, they seem to not comprise the same specificity. Biosynthetic cluster from radicicol, which has been isolated from some fungi containing putative halogenases (Cdr2 or radH), assisted to define how these enzymes can act in different metabolite cores [27,28]. Specific halogenating enzymes for C-8 and C-2 sites from tryptophan have already been exploited either. The enzymes RebH and CmdE, responsible for the halogenation at C-8 and C-2, respectively, in rebeccamycin and chondramida-D, are the main representatives for this type of reaction but still only better clarified in bacterial species [29,30].

Aiming to prove the unambiguous microbial origin of the new haloalkaloids isolated from *T. wortmannii* and verify if their biosynthesis is potentially conducted by FADH_2_ dependent halogenases, the strain was submitted to genomic analysis. The comparison from the reported data with amino acids sequences of halogenases produced by eukaryotic organisms, allowed the identification of four conserved and appropriate regions for the construction of four degenerate PCR primers (NCBI database). Using such primers over four combinations of genomic DNA from *T. wortmannii* as a template, a fragment of approximately 1134 kbp could be amplified in a combination of J5 and J7 primers (Section 3.10). The sequencing and analysis of the PCR product of 1.1 kbp showed identity between 35% and 45% halogenases described in NCBI database, from eukaryotic organisms. According to these data, the most outstanding result was obtained with the similarity alignment with radH halogenase amino acid sequence from *Aspergillus oryzae*, as it is also displayed in Figure S50. Despite the low identity in terms of percentage between the radH halogenase and the amino acid sequence obtained from *T. wortmannii*, the result still prints evidences concerning the occurrence of encoded halogenase in the genome of the fungus. Apparently, some regions of the PCR fragment sequence have introns, which may be one reason for the low level of similarity. Nevertheless, the GWXWXIPL region (Figure S50) is specific for confirming halogenases and considered to be a conserved region for all dependent halogenases of FADH_2_ [31]. This preliminary study did not enable the inference that halogenations in C-2 and C-8 result from the activity of the same enzyme or perhaps from two different halogenases. However, the described results evince that the halogenation of the rugulovasines A and B precursors potentially occur by an enzymatic process. Based upon the detection of the amplified fragment described above, the ongoing work with the strain has focus on the isolation and the enzymatic activity of the halogenase to corroborate with the biosynthetic studies of the halorugulovasines from *T. wortmannii*.

### 2.4. Antibacterial Assay

The isolated compounds 8-chlororugulovasine A, 8-chlororugulovasine B and the new compound 2,8-dichlororugulovasine A were tested against gram-negative bacteria *Escherichia coli* ATCC 25922. Any meaningful response was observed for the tested concentrations. The obtained MIC for the tetracycline (used as control) was 3.9 μg/mL.

## 3. Experimental Section

### 3.1. Isolation, Identification, and Preservation of T. wortmannii

The fungal strain was isolated from a sample of apple fruits (*Malus domestica*, Gala cultivar) purchased at a local market in São Carlos (São Paulo state, Brazil—January 2008). The fruits were washed with liquid soap under tap water and subjected to an adapted methodology for isolation of endophytes [21]. Disinfection of fruit surface was done by alternating immersions in 70% EtOH solution, H_2_O, 11% aq. NaClO solution, and H_2_O, for 1 min each. The fruits were then aseptically peeled, and fragments of pulp were transferred to Petri dishes containing potato dextrose agar (PDA, formulation in [32]) and incubated in the dark at 25 °C for one week. The *Talaromyces* strain grew from one pulp fragment and was purified by re-streaking on PDA. It produced restrict colonies of yellowish mycelium covered with masses of green conidia on standard mycological media (Figure S51). Microscopically, it produced biverticillate conidiophores having acerose phialides and spherical to ellipsoidal conidia, characteristic of the genus *Talaromyces* [33]. It was identified as *Talaromyces wortmanii* by sequencing of a fragment of the second major subunit RNA polymerase (RPB2) and comparison with reference sequences of *Talaromyces* species deposited in the Genbank [34]; followed by a phylogenetic analysis of Maximum Likelihood (Figure S52) conducted with Mega software [35]. The generated DNA sequence was deposited in the GenBank, accession number KT004676. The strain is currently preserved at −80 °C in the Coleção Micológica de Lavras (CML), Universidade Federal de Lavras, Lavras, Brazil, under the accession number CML 2704.

### 3.2. Chemicals and Standards

All employed solvents and chemicals were of analytical grade (Sigma-Aldrich, Steinheim, Germany, unless otherwise stated). For HRMS analysis, LCMS grade was used while HPLC grade solvent was applied during metabolites extraction and purification. H_2_O was purified in Milli-Q system (Millipore, Bedford, OH, USA). The used standards of fungal secondary metabolites were obtained from the *in house* microbial collection [9,10].

### 3.3. UHPLC-DAD-QTOF and UHPLC-Orbitrap Analysis

The UHPLC-HRMS based dereplications were carried out in an Ultimate 3000 UHPLC system with DAD detector (Dionex, Sunnyvale, CA, USA) coupled to a maXis G3 quadrupole time-of-flight (QTOF) mass spectrometer (Bruker, Bremen, Germany). The spectrometer was equipped with an electrospray (ESI) source, operated at a resolution of 50,000 full width at half maximum (FWHM), with mass spectra recorded in the range 100–1000 Da with 5 scans per second. The acquired spectral data were calibrated using sodium formate automatically infused prior to each analytical run and providing a mass accuracy better than 1.5 ppm. Separation of 1 μL samples were performed at 40 °C on a RP Kinetex C_18_ column (100 × 2.1 mm, 2.6 μm, Phenomenex, Torrance, CA, USA) employing linear gradient of H_2_O/CH_3_CN (90:10 → 0:100, *v*/*v*, both buffered with 20 mM of FA) in 10 min at a flow rate of 0.4 mL/min. UV/Vis spectra were collected at wavelengths from 200 to 700 nm. All micro-extracts were manually dereplicated with assistance of the software DataAnaysis 4.0 software (Bruker). The UHPLC-MS/HRMS analysis were performed through an Accela High Speed LC coupled to a LTQ Orbitrap Velos FT-MS (ThermoFisher Scientific, San Jose, CA, USA), equipped with an electrospray source (HESI-II) and operated at a resolution of 60,000 FWHM, with positive ionization and 25 eV at MS/HRMS mode. The chromatographic system was fitted with a RP Luna C_18_ column (150 × 4.6 mm, 5 μm, Phenomenex) applying H_2_O/CH_3_CN (80:20 → 0:100, *v*/*v*, both buffered with 20 mM of FA) in 15 min at a flow rate of 0.5 mL/min. The software Thermo Xcalibur 2.1 (ThermoFisher Scientific) was used to control the full system.

### 3.4. LC-UV-SPE-NMR

The LC-UV-SPE instrument comprised an Agilent 1200 series HPLC (Agilent Tecnologies, Santa Clara, CA, USA) equipped with a G1315D DAD detector and a G1329A autosampler. Hystar 2.3 software (Bruker, Bremen, Germany) was used to control the LC system. The chromatography was directly coupled to an automatic cartridge exchanger (Bruker) equipped with a range of cartridges containing different stationary phases, where the flow was directed automatically. The NMR measurements were carried out on a Bruker Avance III instrument (14.1 Tesla/600 MHz, Bruker) fitted to an automatic sample changer and a TCI 5 mm triple resonance (^1^H/^13^C/^15^N) z-field gradient cryo-probe. The probe was fitted with an automatic tuning and matching unit and a Cryo-FIT converter for coupling to the LC-SPE system.

### 3.5. Media Culture and Micro-Extractions

For the screening process, the strain isolated from apple fruits was grown in potato dextrose agar (PDA), creatinine sucrose agar (CREA), malt extract agar (MEA), czapeck yeast autolysate agar (CYA), dichloran rose bengal yeast extract sucrose (DRYES), malt extract agar Oxoid^®^ (MEAox), Wickerhams antibiotic test medium (WATM), yeast extract sucrose agar (YES) and oatmeal agar (OA). For medium formulations, see [32]. The fungus was three-point inoculated in each medium and incubated at 25 °C in the dark for 14 days. Five plugs (6-mm diameter) along the diameter of the fungal colony were cut out and extracted according to the micro-extraction method developed by Smedsgaard [36]. The applied extraction solvent was a mixture of MeOH–CH_2_Cl_2_–EtOAc (1:2:3 *v*/*v*/*v*) with 1% of FA.

### 3.6. Scaled Up and Metabolites Extraction

The PDA medium was chosen for the large-scale cultivation of *T. wortmannii*. Nevertheless, the fungal incubation on solid medium was switched to the growth within PD (potato/dextrose) liquid medium (Difco, Lawrence, PA, USA). pH = 5.6 was reached by using HCl solution (0.1 mol/L). Three PDA plugs from the strain (1 cm^2^) were aseptically transferred 30 Erlenmeyer flasks (500 mL) containing 125 mL of PD medium, previously sterilized for 15 min, 121 °C. After 14 days at 25 °C, the mycelium was separated by reduced pressure filtration and the liquid phase was submitted to liquid-liquid fractionation with EtOAc (3 × 500 mL). The mycelia were combined and crushed altogether with MeOH (4 × 500 mL). The extraction was kept during 12 h in the dark, under static conditions with following vacuum filtration. The crude extracts from the EtOAc and MeOH extraction were obtained separately after drying. However, as the compounds of interest were detected in both crude extracts, they were then combined. The micro-extraction mixture of solvents was not applied during the extraction process in order to avoid the appearance of potential halogenated artifacts.

### 3.7. Metabolites Purification

The PD crude extract was subjected to CombiFlash chromatography system (Teledyne Isco, Lincoln, NE, USA), fitted to a RP C_18_ column (100 g, 87.7 mL, Teledyne Isco), after which the linear gradient of H_2_O/MeOH (70:30 → 0:100, *v*/*v*) was applied in 20 min with the flow rate of 40 mL/min. This process provided the fractions Fr1–Fr15. The fractions Fr8 and Fr9 were combined and refractionated on RP C_18_ column (30 g, 26.4 mL, Teledyne Isco) using the gradient H_2_O/MeOH (60:40 → 0:100, *v*/*v*) in 25 min with flow rate of 20 mL/min, which afforded the subfractions Fr16–Fr28. The subfraction Fr20 was injected in a preparative HPLC SIL-20AP VP (Shimadzu, Kyoto, Japan) with phenyl–hexyl column (250 × 21.20 mm, Phenomenex) applying the gradient of H_2_O/ACN (70:30 → 40:60, *v*/*v*) in 20 min, flow rate 14 mL/min. The samples Fr29–Fr40 were obtained and the sample Fr32 showed to be enriched in the halocompounds whose yield was 2.5 mg. This amount was then dissolved in 1.5 mL of H_2_O/MeOH (*v*/*v*) and filtered through a PVDF membrane syringe filter prior to the injection into the LC-UV-SPE-NMR system.

The chromatographic separation was performed on a Luna C_18_ column (150 × 4.60mm, 5 µm, Phenomenex) with the gradient elution of linear gradient of H_2_O/ACN (80:20 → 40:60, *v*/*v*, both buffered with 5 ppm of TFA) in 20 min with a flow rate of 1.0 mL/min. The peaks of interest were detected through the UV absorbance at 285 nm and adsorbed on SPE cartridges (HySphere Resin GP-polydivinylbenzene, 10 mm × 2 mm, 10 μm). Thirty consecutive chromatographic runs were performed, with volume injections of 20 μL. After the trapping process, the cartridges were dried with nitrogen for 30 min to remove residual solvent. Methanol-*d*_4_ (99.8% D) was used to elute the compounds from the SPE cartridges directly into 3 mm NMR tubes.

### 3.8. Investigation of Brominated Compounds Produced by T. wortmannii

In order to verify the potential biosynthesis of brominated compounds by the microorganism two different experiments were carried out in triplicate. The first test was performed through the use of HBr solution (0.01 mol/L) instead of HCl for pH correction of the liquid culture medium (pH = 5.6). The second experiment comprised the addition of KBr salt at the concentration of 0.2 mg/L into the fermentation medium with no buffer use. The strain incubation and the crude extract was performed as established in Section 3.6. The triplicate samples were redissolved in methanol (0.1 mg/mL), filtered and subjected to LC-MS (2 µL injection vol.) or alternatively to UHPLC-HRMS analysis (0.2 µL injection vol.).

### 3.9. Antibacterial Assay

The activity assay was performed against *E. coli* ATCC 25922 applying microbroth dilution assay as recommended by Clinical Laboratory Standards Institute *CLSI* (former *NCCLS*) [37]. The tested bacteria were incubated in the MH broth. The assays were performed on 96-well plates, in triplicate for each tested compound, in the concentrations of 250, 125, 62.5, 31.2, 15.6, 7.8 and 3.9 µg/mL. Bioactivity was recorded as blue coloration in the wells accordingly the cells viability after resazurin dye reaction. Tetracycline was used as positive control and pure DMSO (Merck, Darmstadt, Germany) as negative control.

### 3.10. PCR Amplification

Degenerated oligonucleotides were designed to locate the halogenase gene in total DNA of *T. wortmannii*. They were derived from four conserved motifs present in the amino acid sequences of known eukaryotic halogenases proteins. These oligonucleotides were used to attempt PCR amplification using total DNA from *T. wortmannii* as the template. The sequences of two sense oligonucleotides were J5 5′-GTGGTTGGTGGTGGCCCTGGAGGG-3′, J6 5′-TCGACCGCTGGCATCGACCAA-3′, J7 5′-TGGGCATGGTTCATTCCTCTCCACAAC-3′ and J8 5′-CCCAGATGAGAAGAATGGGTCAATGAA-3′. PCR mixtures contained 50 ng of template DNA, 50 pmol of each primer, 1 mM MgCl_2_, 2.5 U Taq DNA Polymerase (MBI Fermentas), 2.5% DMSO and 0.2 mM dNTP-mix. Amplification was obtained by standard procedure with an annealing temperature of 50 °C.

## 4. Conclusions

UHPLC-HRMS based dereplication conducted the detection of unknown halocompounds biosynthesized by *T. wortmannii*, a fungus isolated from symptomless apple fruits. The 2,8-dichlororugulovasine A and B were purified from the fungal growth in PD medium while the 2-bromorugulovasine A and B were observed as co-products when inorganic additives were used into the incubation medium. The dichlorinated compounds had the structural elucidation supported by NMR 1D/2D spectroscopy combined to accurate full-scan HRMS and MS/HRMS data, while the bromine derivatives were identified exclusively by means of thorough tandem MS/HRMS data analysis.

The likely occurrence of FADH_2_ dependent halogenases in *T. wortmannii* enzymatic system was also proposed based on the detection of one conserved region for halogenases within the organism gene code. Halogenation in secondary metabolites produced by fungi occurs mostly in phenolic substrates, although tryptophan had been encountered as free substrate for halogen incorporation for instance in 8-chlororugulovasines from *Penicillium islandicum* [17] and some brominated roquefortines from *Penicillium chrysogenum* [38].

The reaction at indol system is well known in prokaryotic organisms whereas particularly at C-2 from tryptophan core is uncommon either in bacteria or fungal species. As a whole, halogenations seem to be less expressed within fungal enzymatic systems or perhaps less exploited due to the access to microorganisms, which encode such enzymes. In Antibase 2012 [22] for example, there are 22 entries for fungal compounds derived from tryptophan, which own one or more chlorinated sites, while only three hits are encountered for brominated indol moiety and any case is included involving fluorine or iodine as substituents. In view of these observations, the production of the new chlorinated and brominated alkaloids by *T. wortmannii* is a meaningful case to be further explored, taking into account the elucidation of biosynthetic pathways involving biohalogenations from filamentous fungi and mainly to boost the discovery of other untapped halometabolites from terrestrial microorganisms.

## Supplementary Materials

Supplementary materials can be accessed at: http://www.mdpi.com/1420-3049/20/09/17627/s1.
