# Supplementary material for: Dichlorinated and Brominated Rugulovasines, Ergot Alkaloids Produced by Talaromyces wortmannii
Source: Molecules. 2015 Sep 23;20(9):17627–44. doi: 10.3390/molecules200917627 (PMC6332237; doi:10.3390/molecules200917627)
Supplement: Supplementary file 1 [file molecules-20-17627-s001.pdf]

Supplementary Materials

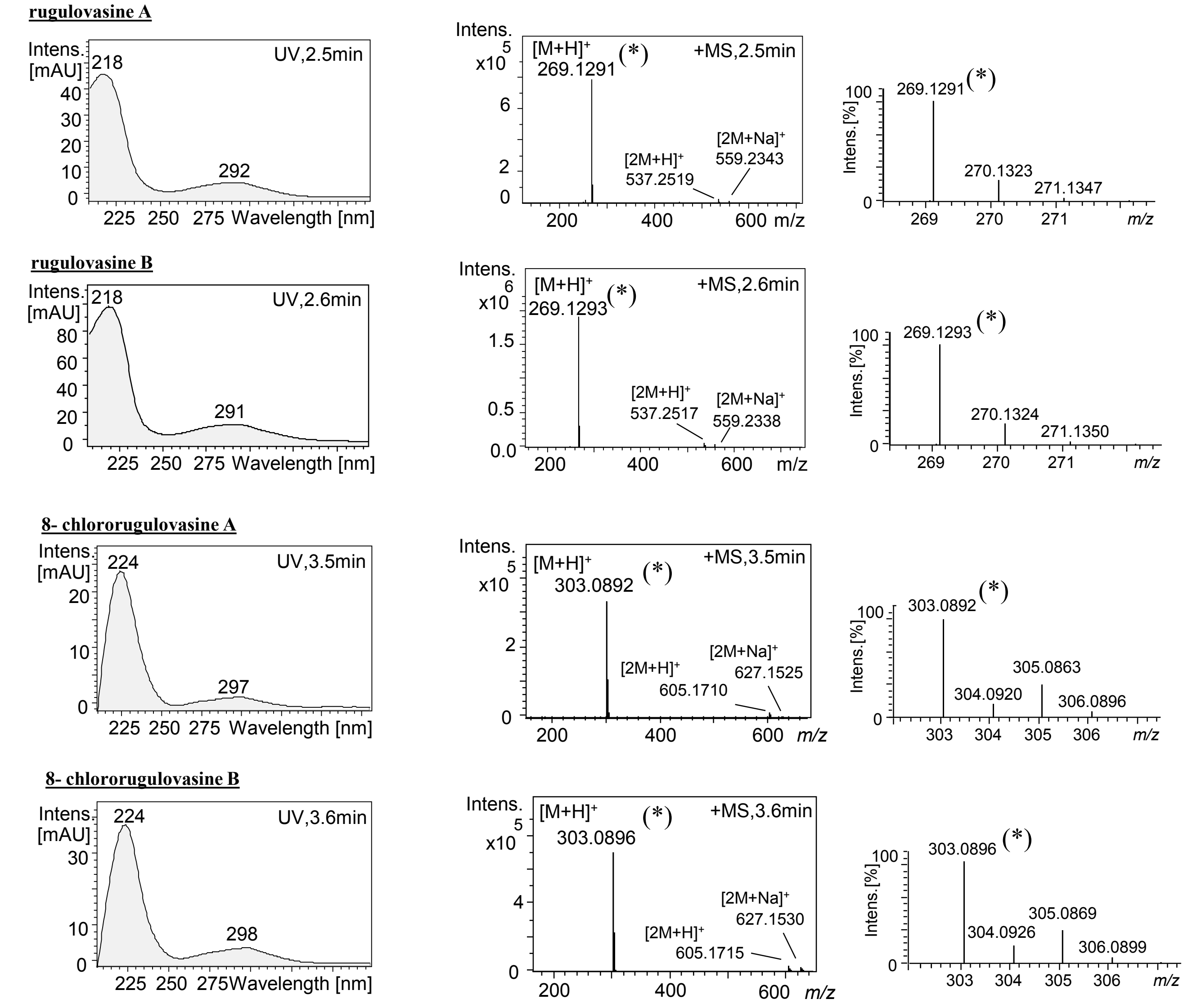

Figure S1. UV and HRMS *full scan* spectra from rugulovasine A and B, 8-chlororugulovasine A and B as indicated. Data obtained from micro-extract of *Talaromyces wortmannii* in PDA medium. (*) Magnified region from the peaks of indicated pseudomolecular ions $[M + H]^+$ and corresponding isotopes.

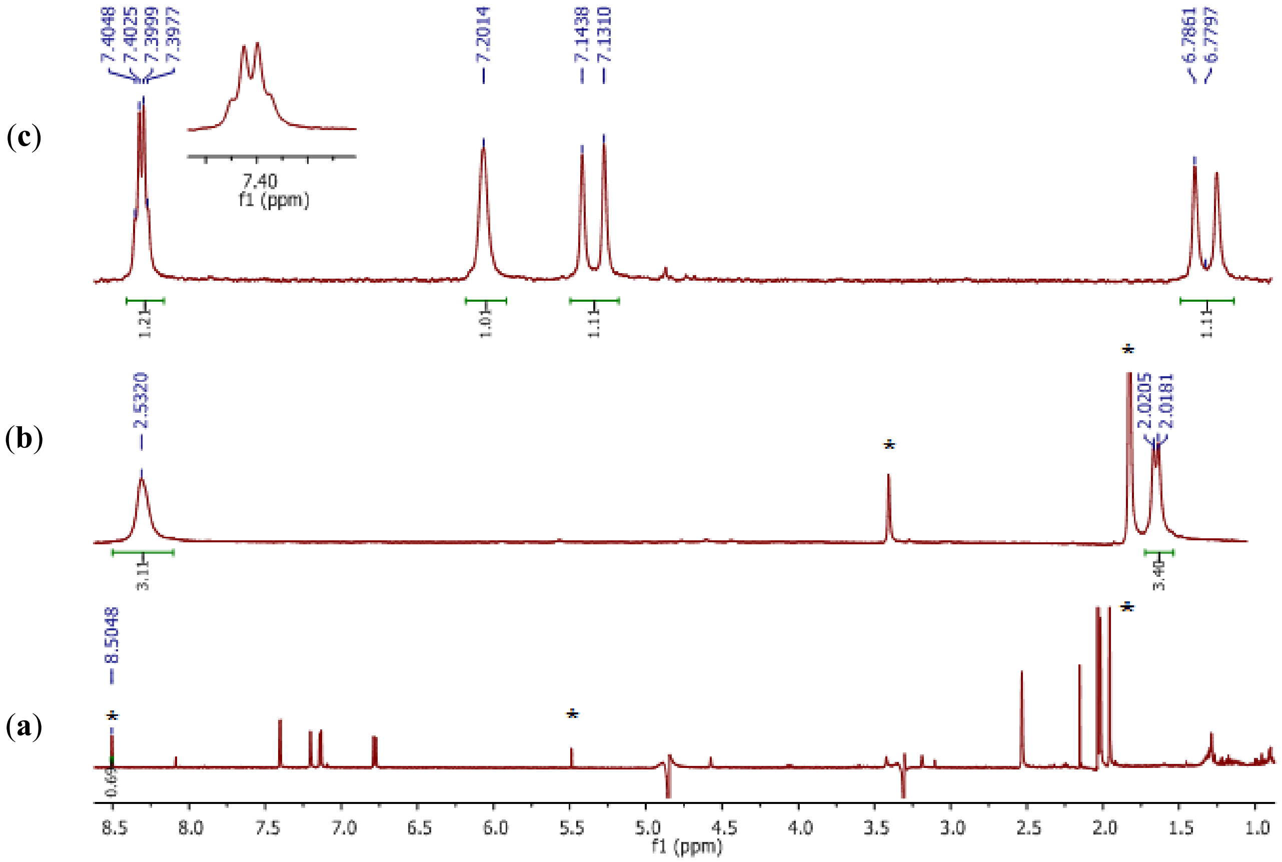

* SPE-cartridge interferents

Figure S2. (**a**) ^{1}H-NMR spectrum of the known pure compound 8-chlororugulovasine A (600 MHz, methanol-d_4); (**b**) magnified region in the range δ_H = 6.7 ppm to δ_H = 7.5 ppm; and (**c**) magnified region in the range δ_H = 2.0 ppm to δ_H = 2.6 ppm. Highlighted numbers under the peaks indicate the integration values from each signal.

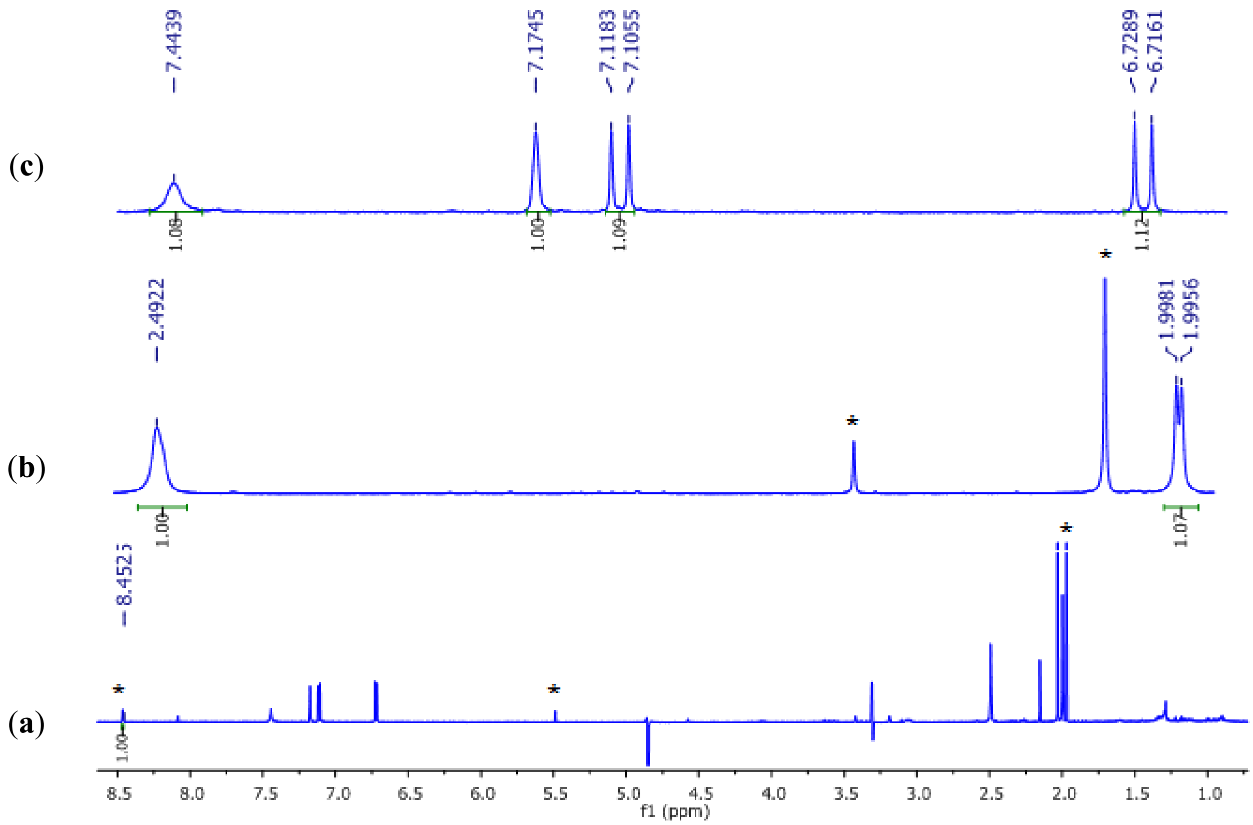

* SPE-cartridge interferents

Figure S3. (**a**) ^{1}H-NMR spectrum of the known pure compound 8-chlororugulovasine B (600 MHz, methanol-d_4); (**b**) magnified region in the range δ_H = 6.7 ppm to δ_H = 7.5 ppm; and (**c**) magnified region in the range δ_H = 1.9 ppm to δ_H = 2.5 ppm. Highlighted numbers under the peaks indicate the integration values from each signal.

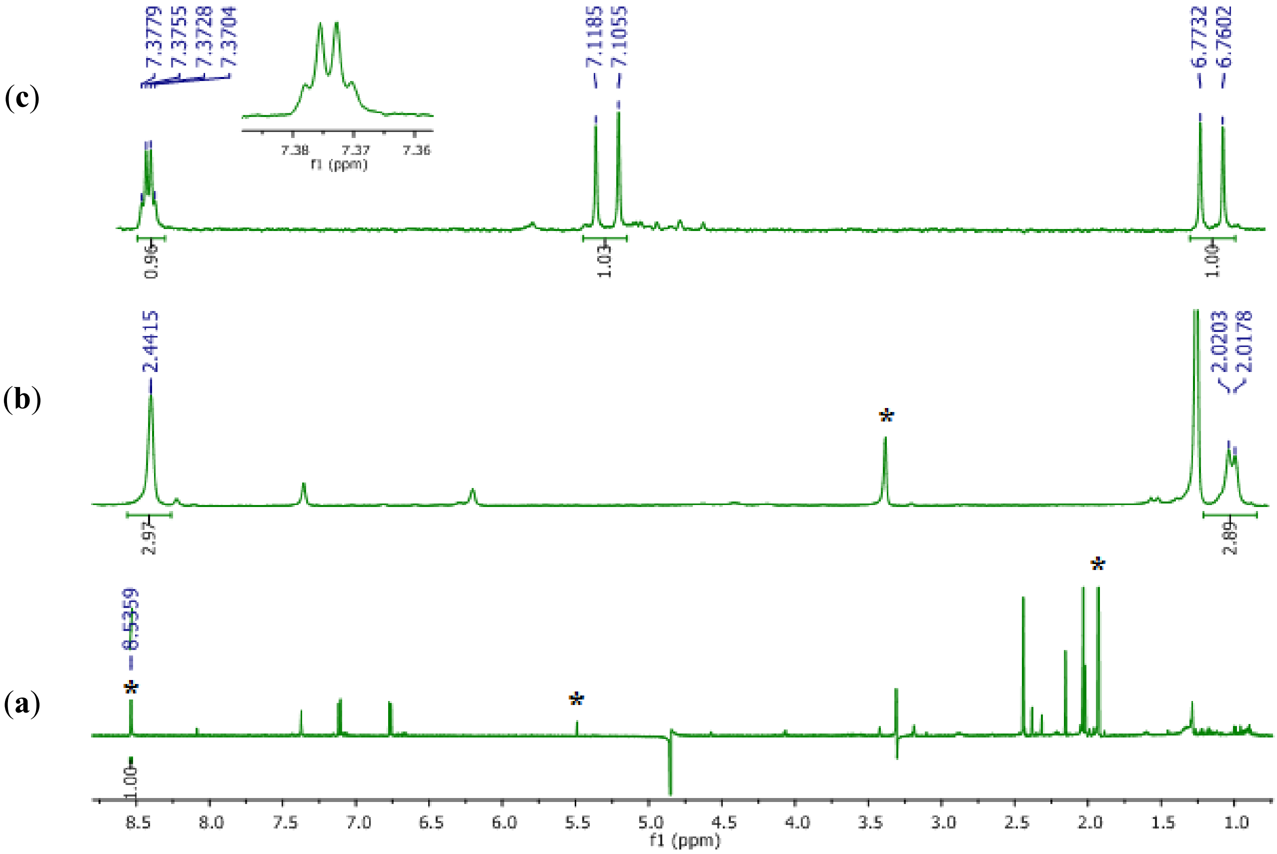

* SPE-cartridge interferents

Figure S4. (**a**) ^{1}H-NMR spectrum of the pure compound 2,8-dichlororugulovasine A (600 MHz, methanol-d_4); (**b**) magnified region in the range δ_H = 6.7 ppm to δ_H = 7.4 ppm; and (**c**) magnified region in the range δ_H = 2.0 ppm to δ_H = 2.5 ppm. Highlighted numbers under the peaks indicate the integration values from each signal.

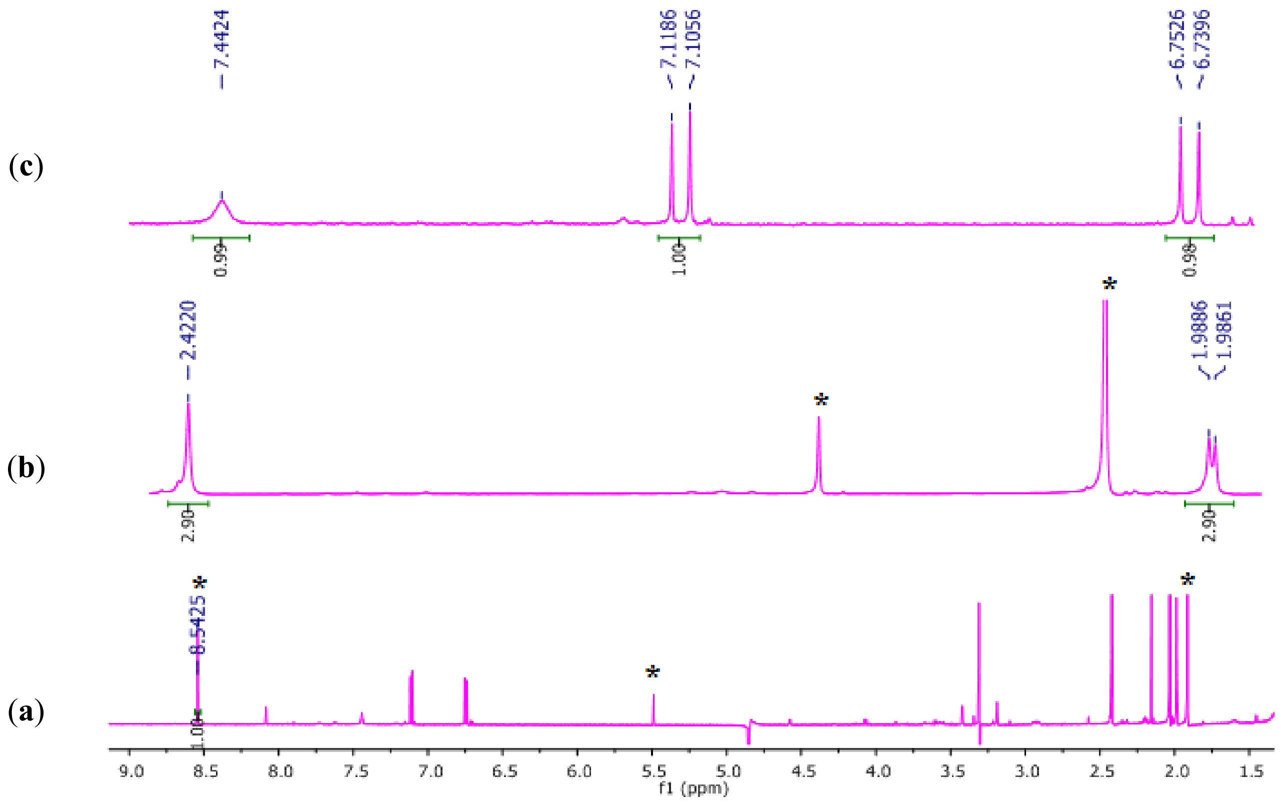

* SPE-cartridge interferents

Figure S5. (**a**) ^{1}H-NMR spectrum of the pure compound 2,8-dichlororugulovasine B (600 MHz, methanol-d_4); (**b**) magnified region in the range δ_H = 6.7 ppm to δ_H = 7.4 ppm; and (**c**) magnified region in the range δ_H = 1.9 ppm to δ_H = 2.5 ppm. Highlighted numbers under the peaks indicate the integration values from each signal.

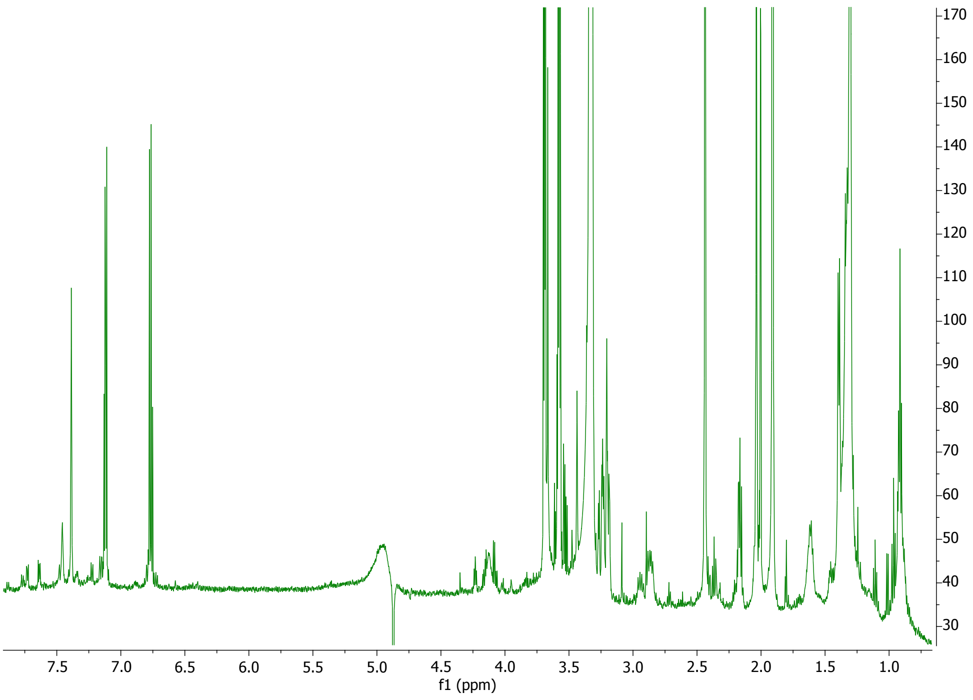

Figure S6. ^{1}H-NMR spectrum from the mixture of compounds 2,8-dichlororugulovasine A and B (600 MHz, methanol-*d*$_4$). 2,8-dichlororugulovasine A represents the major compound within the mixture.

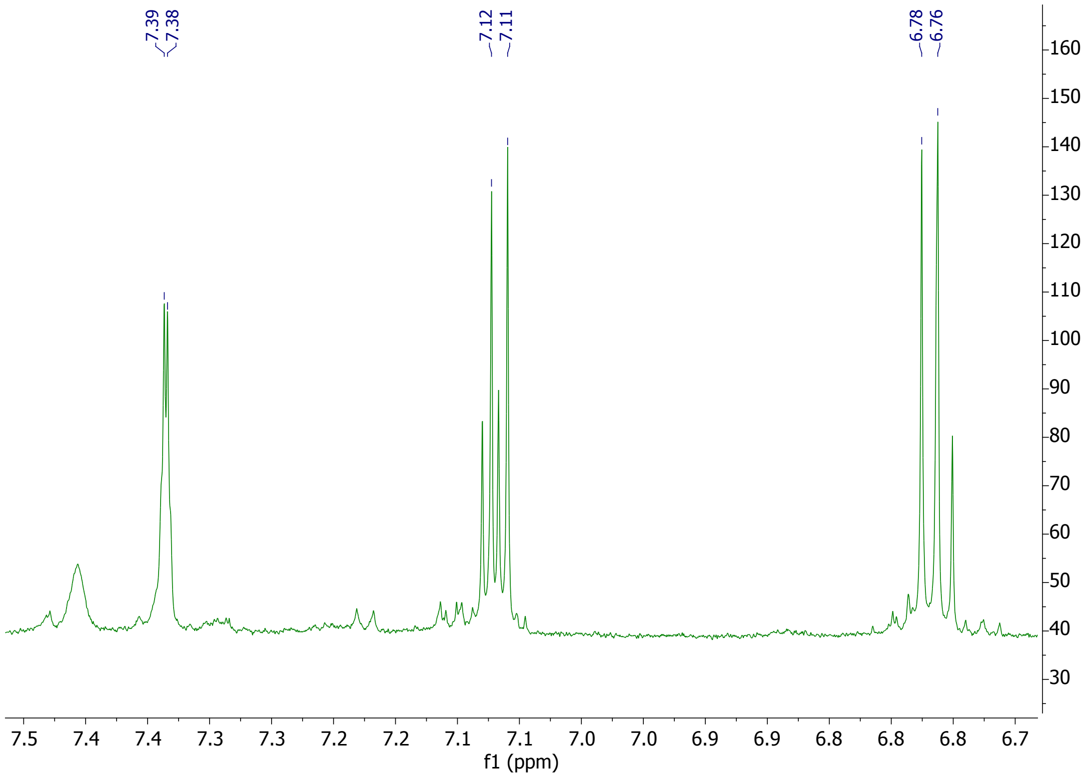

Figure S7. Magnified region from the ^{1}H-NMR spectrum of 2,8-dichlororugulovasine A and B mixture, in the range δ_H = 6.7 ppm to δ_H = 7.5 ppm. 2,8-dichlororugulovasine A represents the major compound within the mixture.

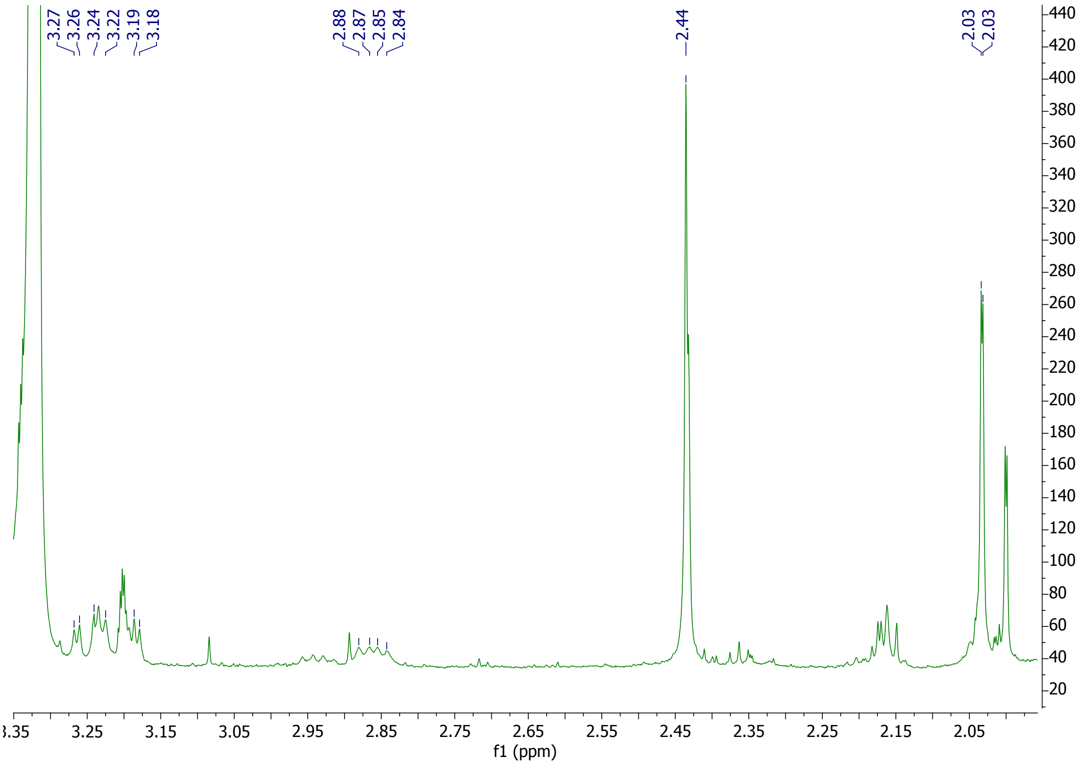

Figure S8. Magnified region from the ^{1}H-NMR spectrum of 2,8-dichlororugulovasine A and B mixture, in the range δ_H = 3.3 ppm to δ_H = 2.0 ppm. 2,8-dichlororugulovasine A represents the major compound within the mixture.

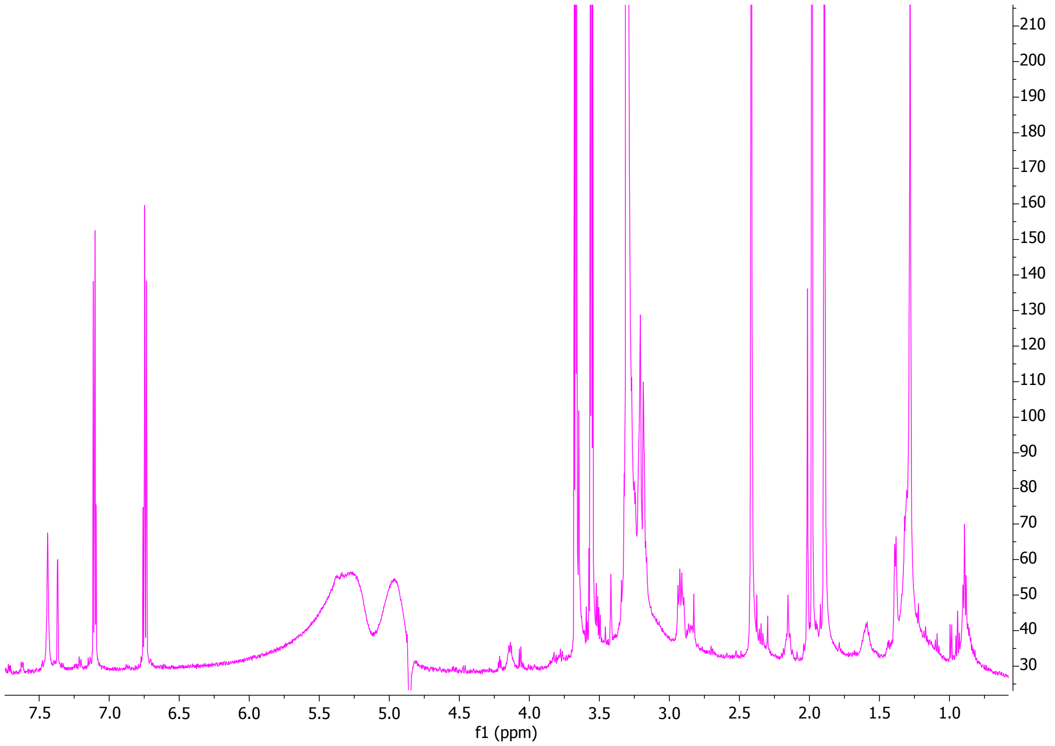

Figure S9. ^{1}H-NMR spectrum from the mixture of compounds 2,8-dichlororugulovasine A and B (600 MHz, methanol-d_4). 2,8-dichlororugulovasine B represents the major compound within the mixture.

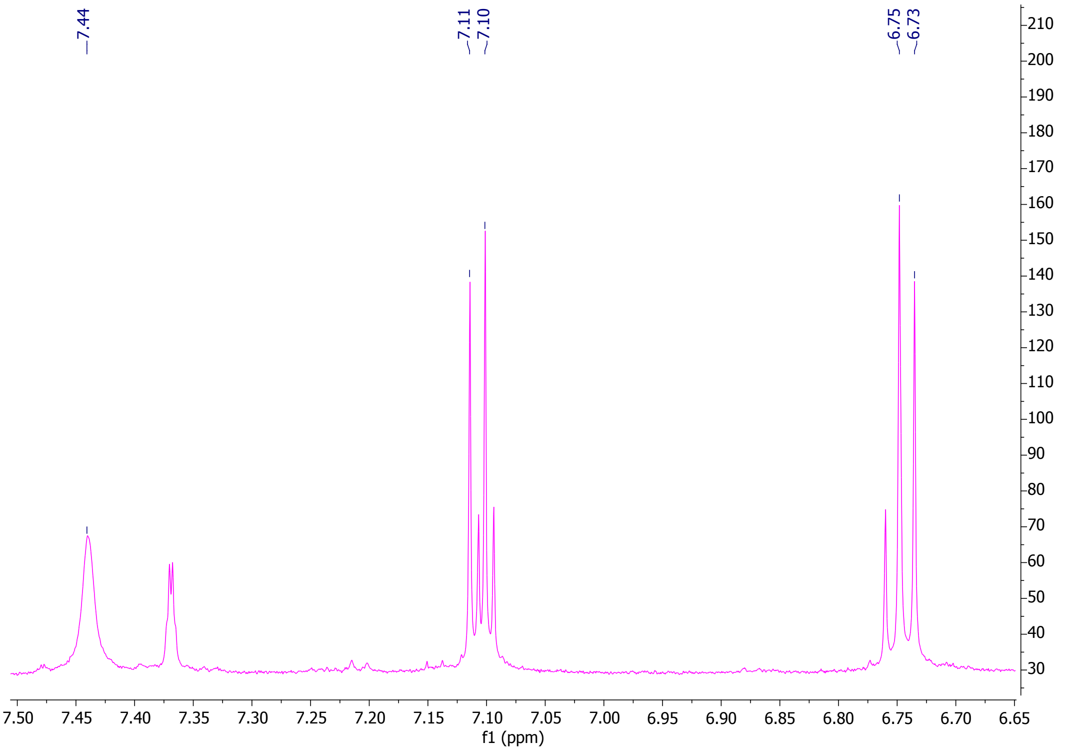

Figure S10. Magnified region from the ^{1}H-NMR spectrum of 2,8-dichlororugulovasine A and B mixture, in the range δ_H = 6.6 ppm to δ_H = 7.5 ppm. 2,8-dichlororugulovasine B represents the major compound within the mixture.

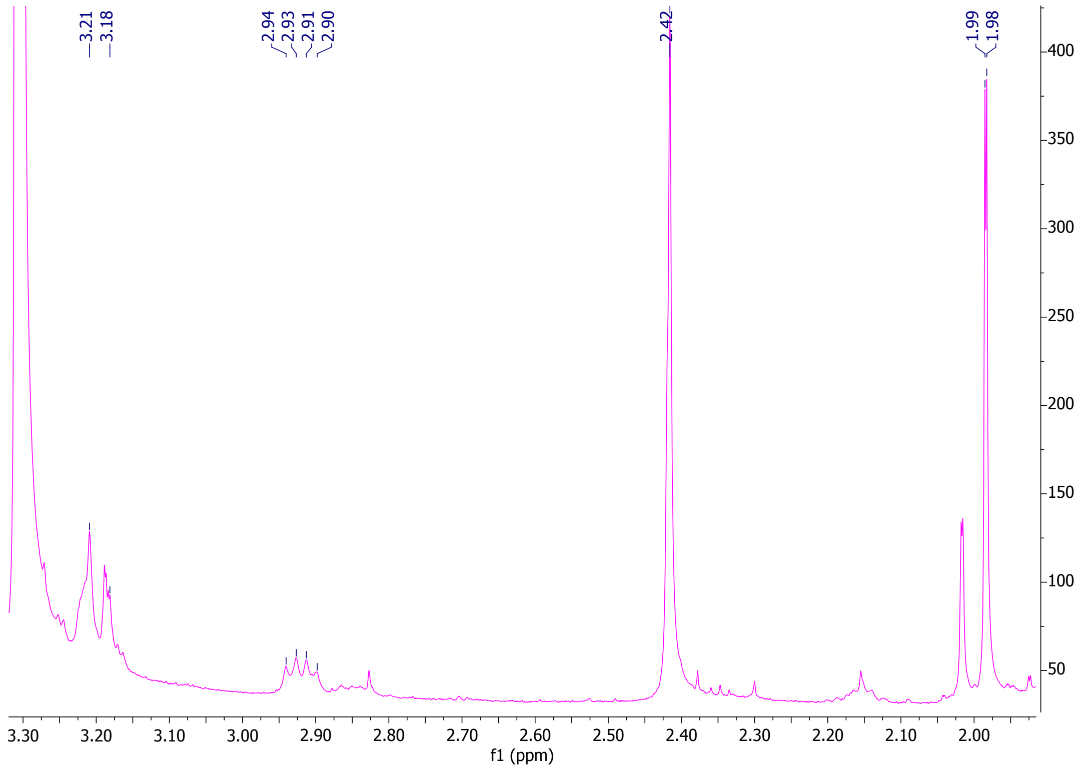

Figure S11 Magnified region from the ^{1}H-NMR spectrum of 2,8-dichlororugulovasine A and B mixture, in the range δ_H = 3.3 ppm to δ_H = 1.97 ppm. 2,8-dichlororugulovasine B represents the major compound within the mixture.

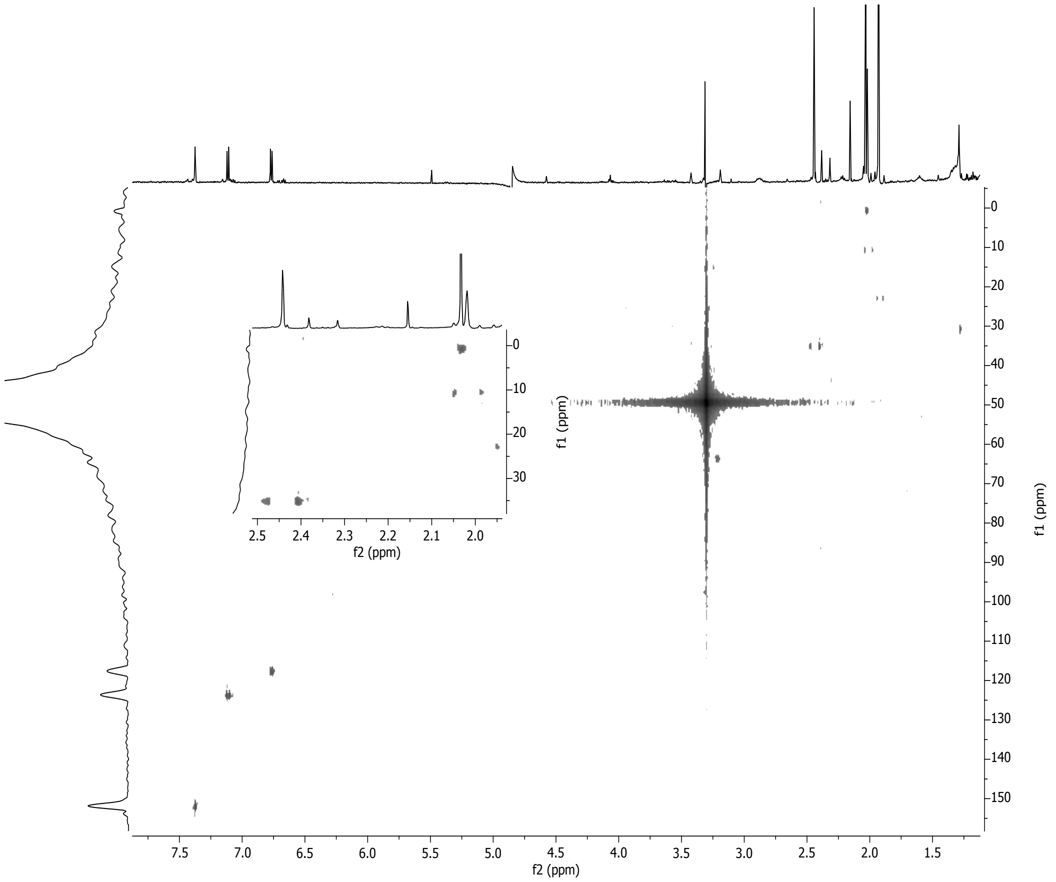

Figure S12. HSQC spectrum of the new compound 2,8-chlororugulovasine A.

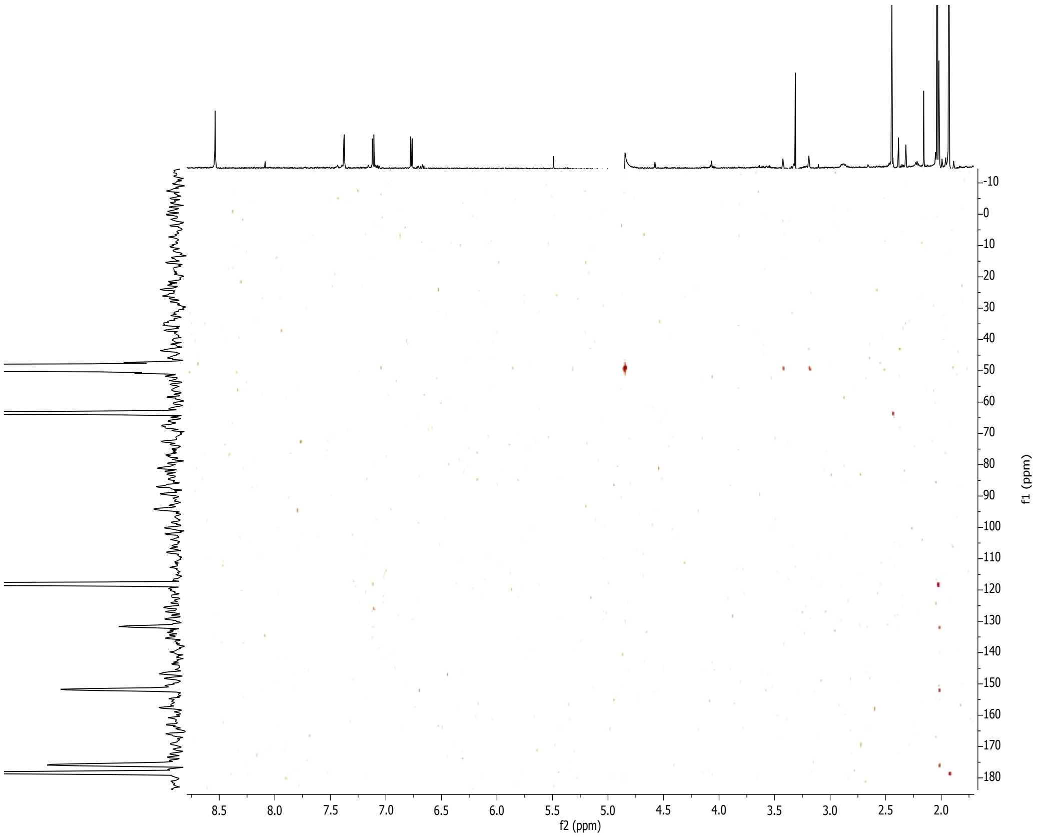

Figure S13. HMBC spectrum of the new compound 2,8-chlororugulovasine A.

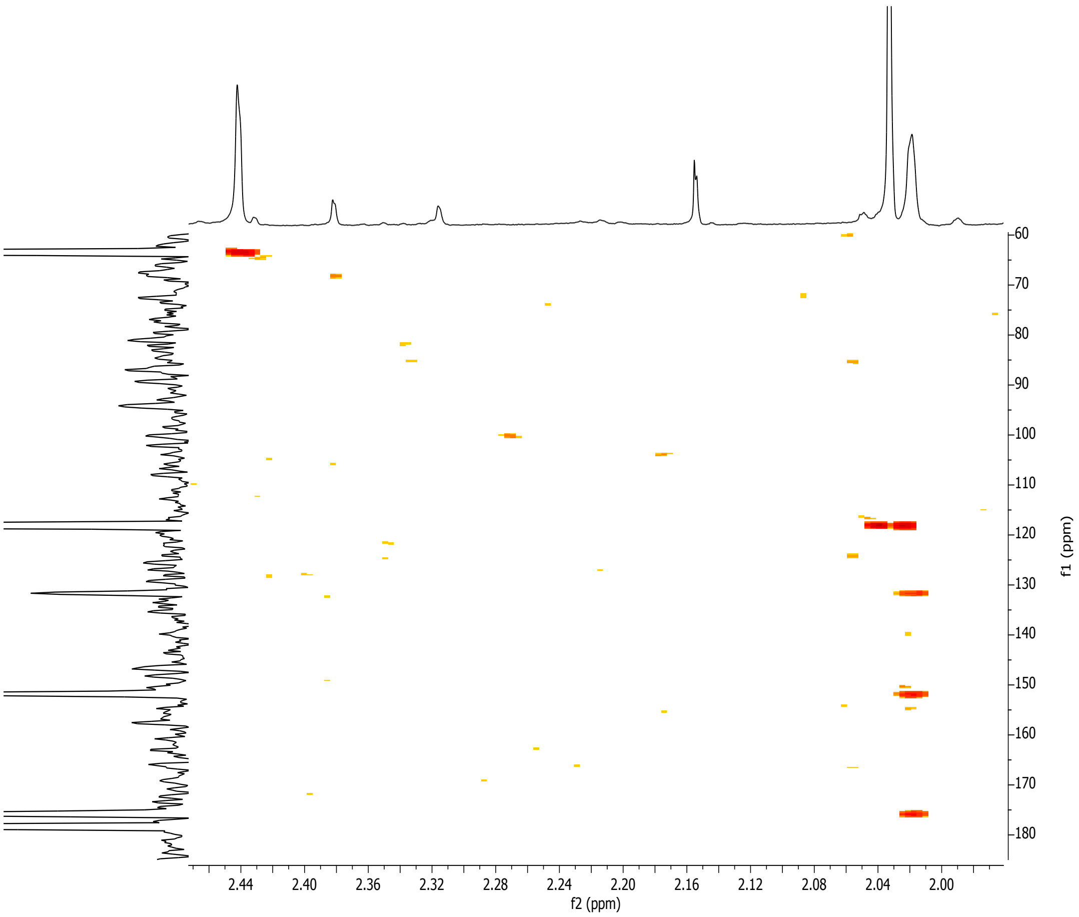

Figure S14. Magnified region from HMBC spectrum of the new compound 2,8-chlororugulovasine A.

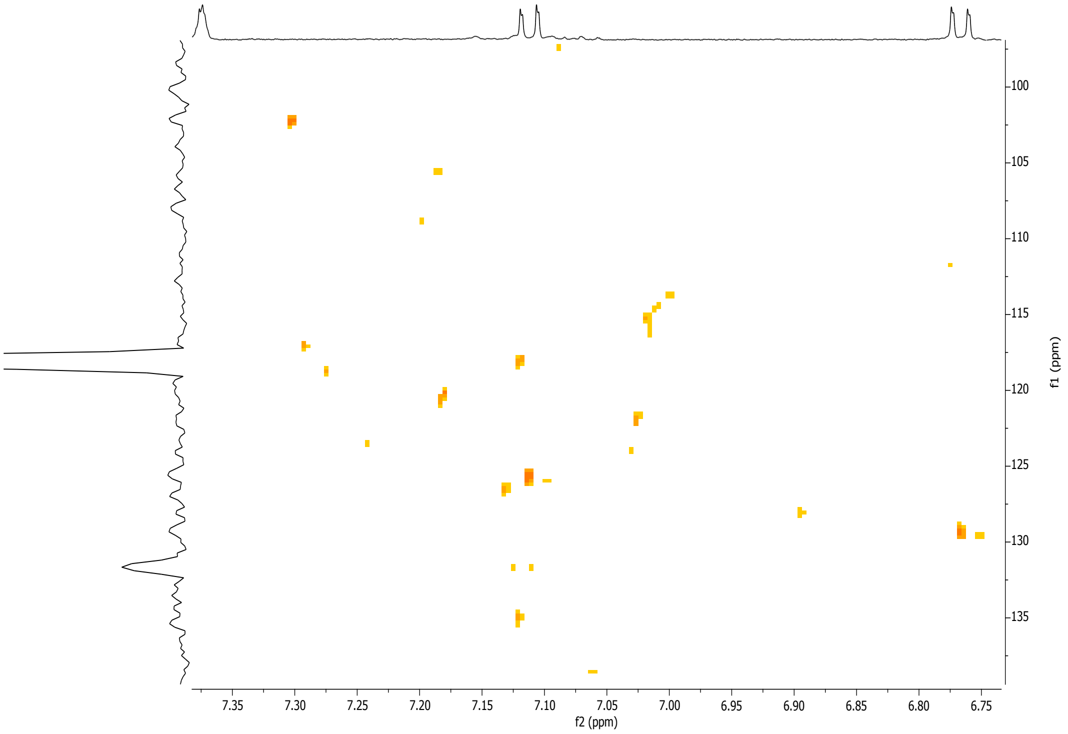

Figure S15. Magnified region from HMBC spectrum of the new compound 2,8-chlororugulovasine A.

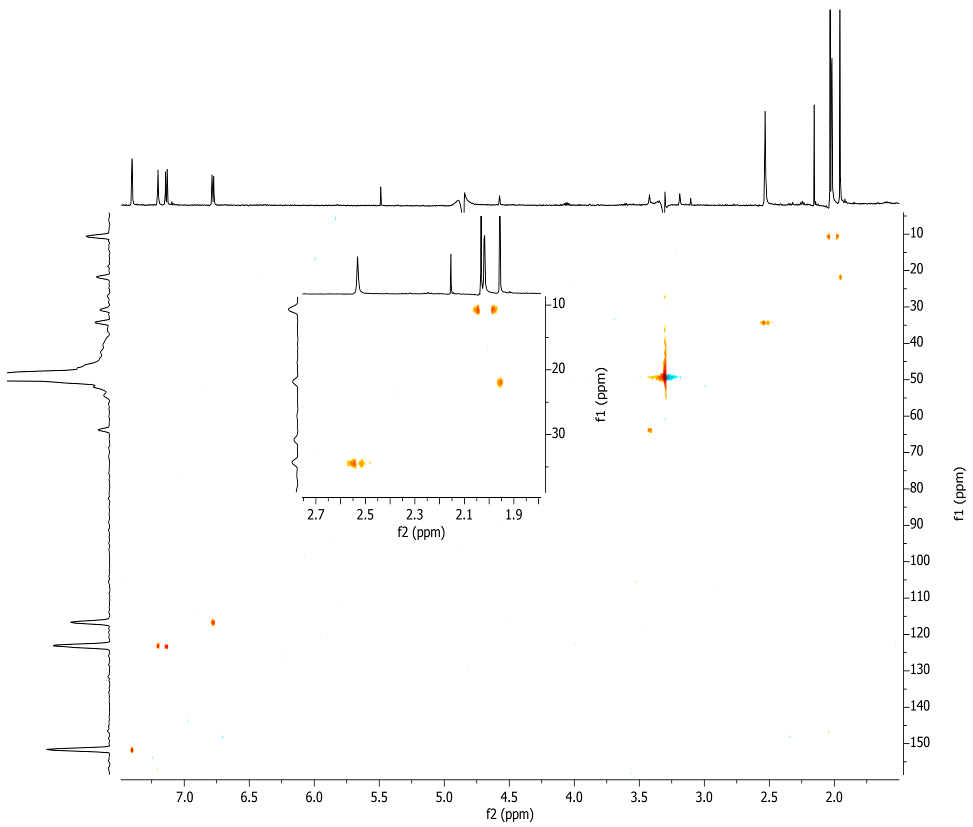

Figure S16. HSQC spectrum and magnified region of the known compound 8-chlororugulovasine A.

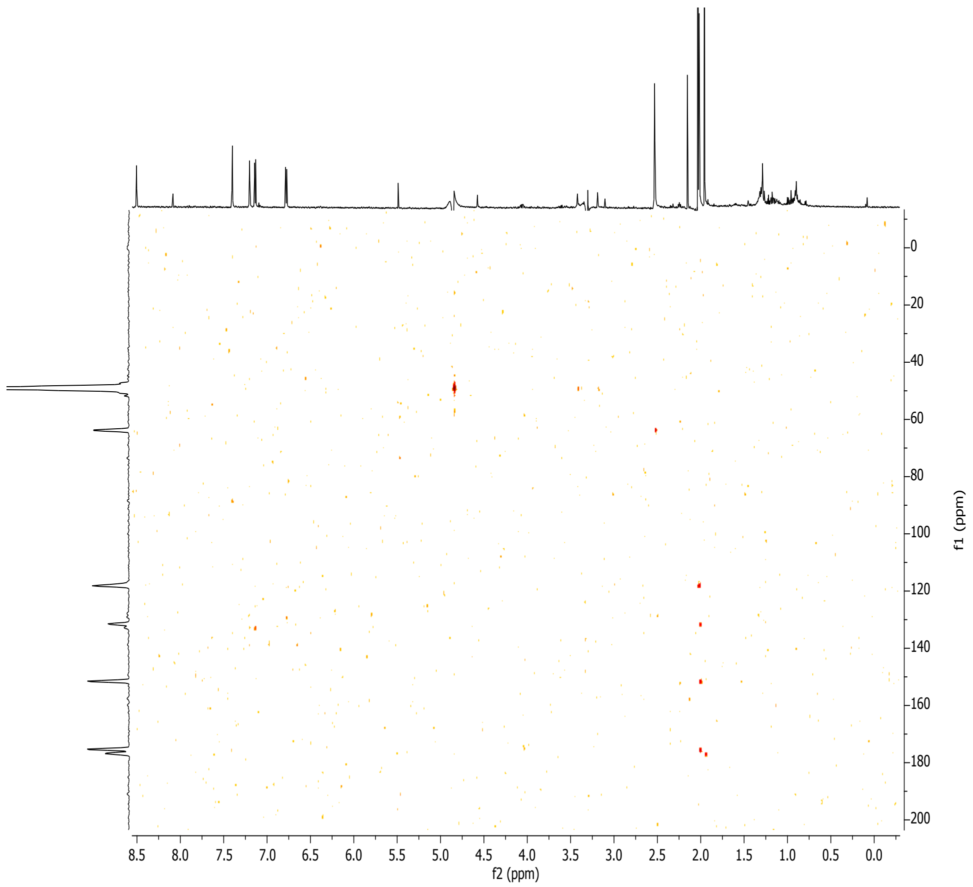

Figure S17. HMBC spectrum and magnified region of the known compound 8-chlororugulovasine A.

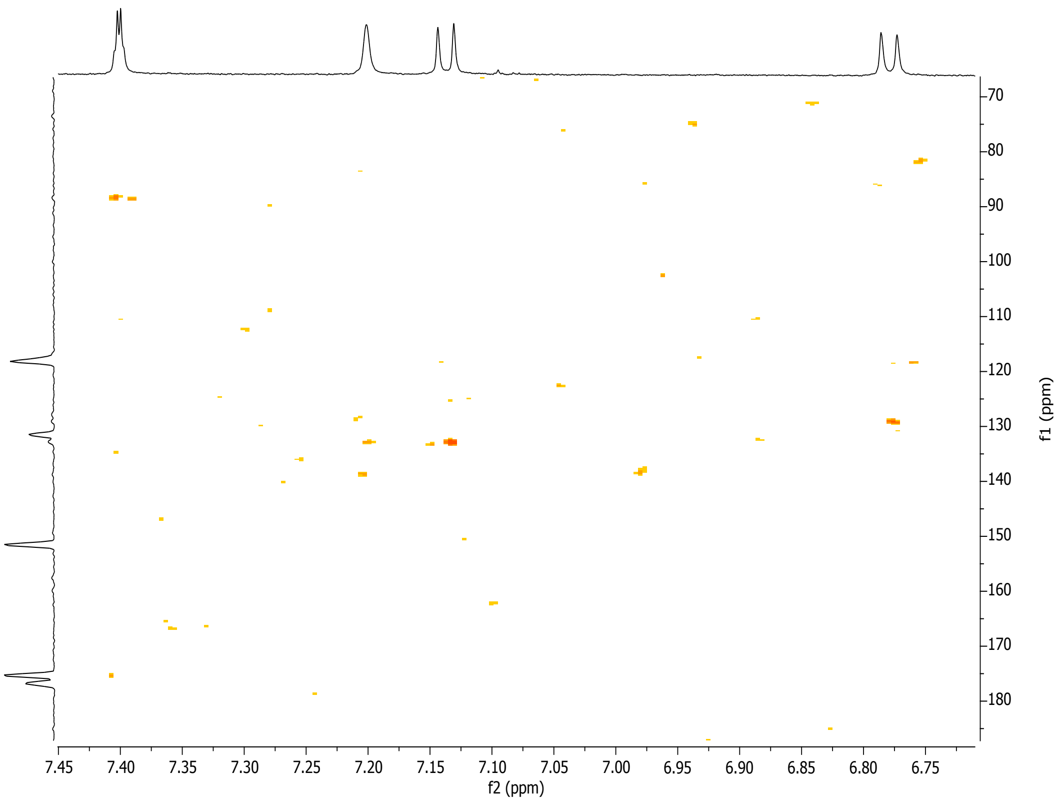

Figure S18. Magnified region from HMBC spectrum of the known compound 8-chlororugulovasine A.

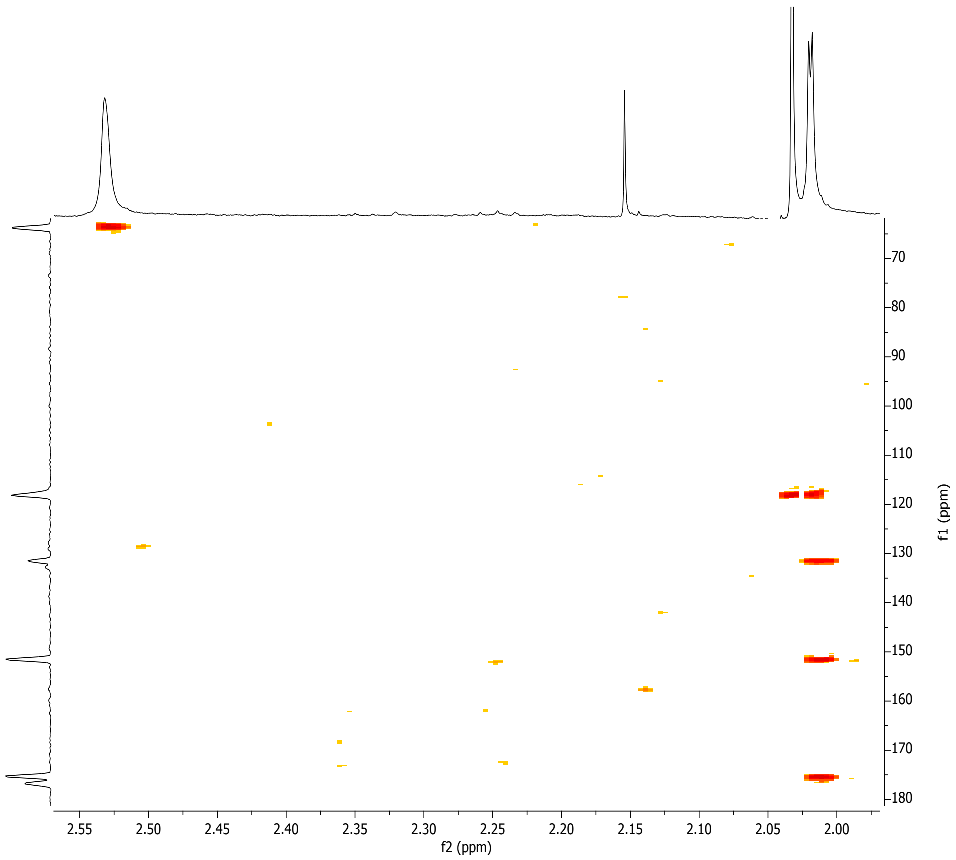

Figure S19. Magnified region from HMBC spectrum of the known compound 8-chlororugulovasine A.

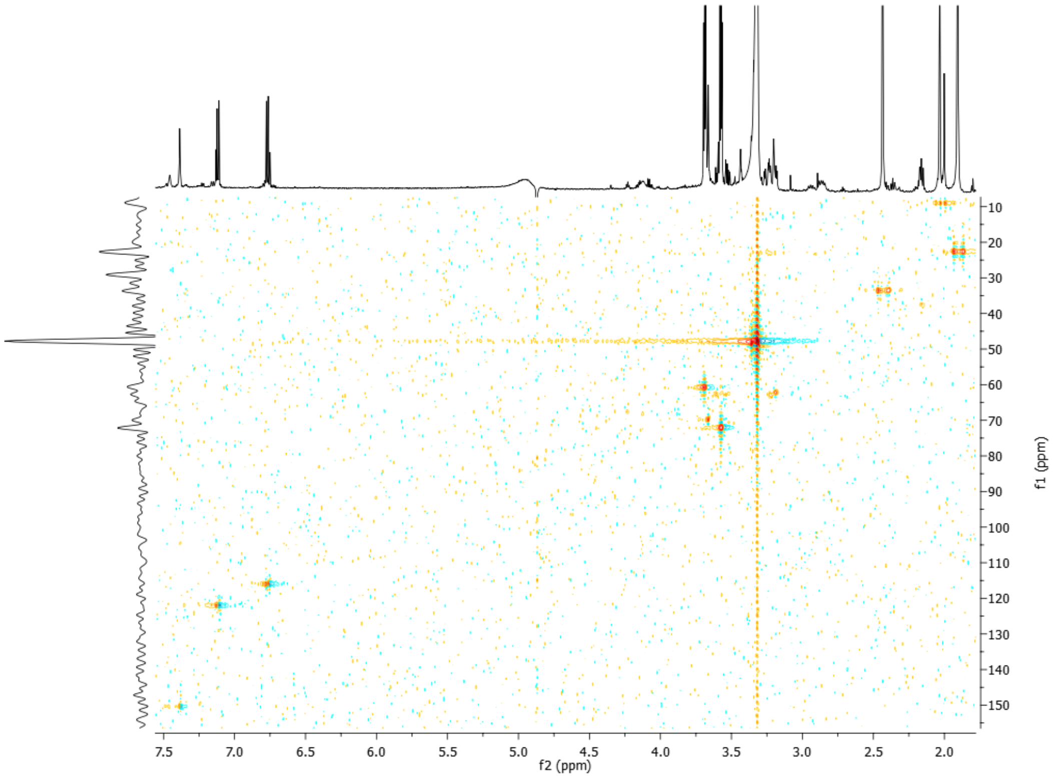

Figure S20. HSQC spectrum from the mixture of 2,8-chlororugulovasine A and B. The isomer 2,8-dichlororugulovasine A represents the major compound within the mixture.

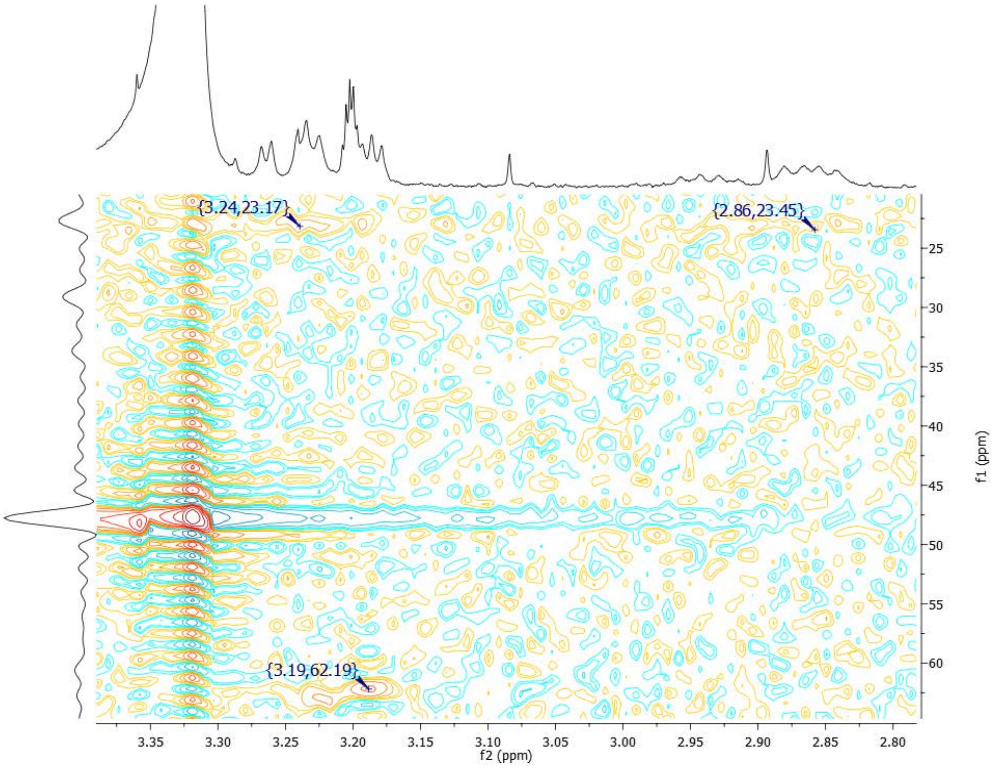

Figure S21. Magnified region of HSQC spectrum from the mixture of 2,8-chlororugulovasine A and B. The isomer 2,8-dichlororugulovasine A represents the major compound within the mixture.

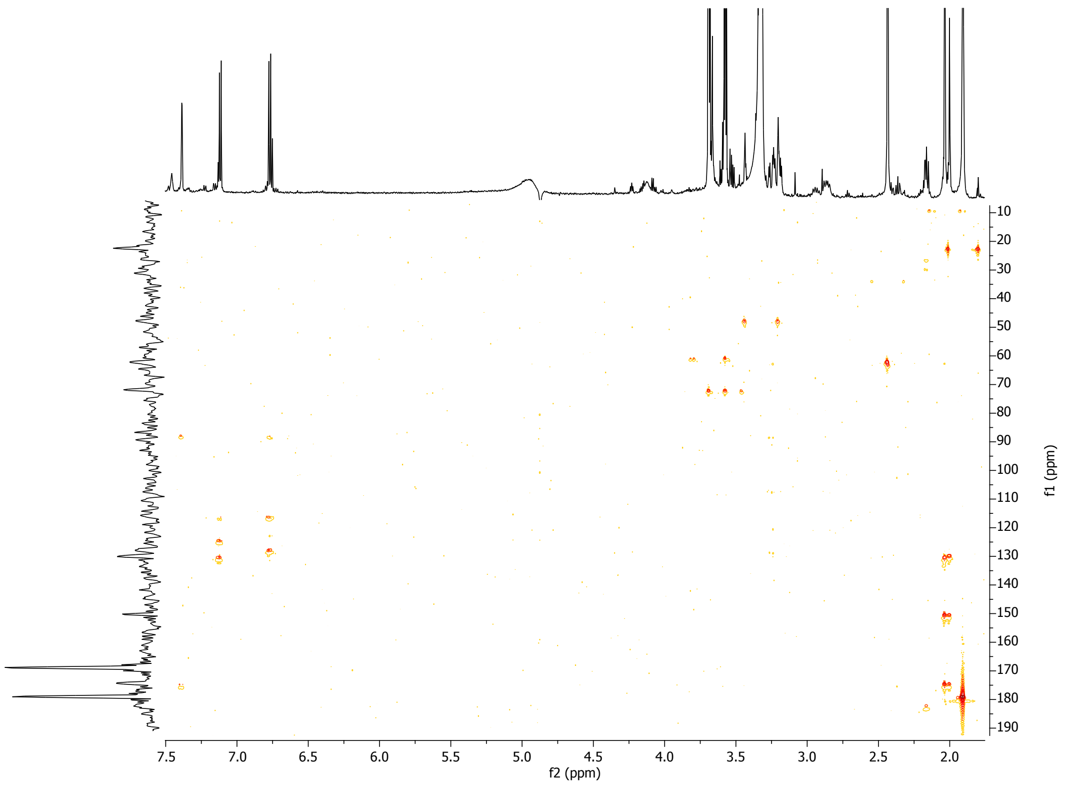

Figure S22. HMBC spectrum from the mixture of 2,8-chlororugulovasine A and B. The isomer 2,8-dichlororugulovasine A represents the major compound within the mixture.

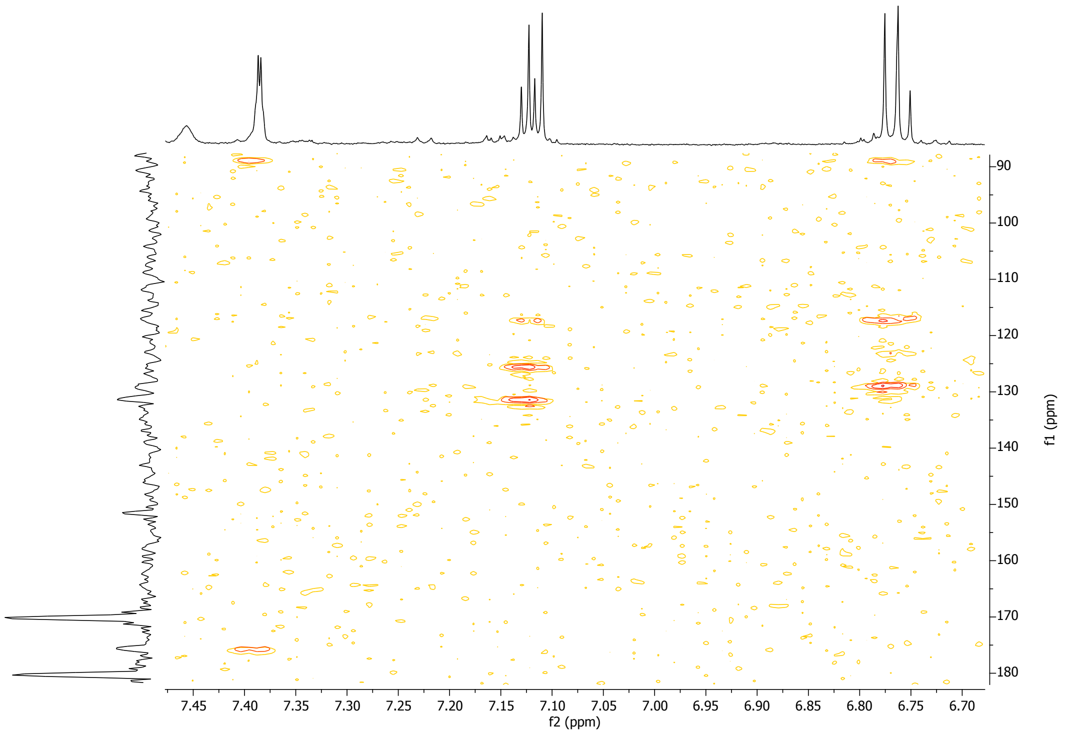

Figure S23. Magnified region of HMBC spectrum from the mixture of 2,8-chlororugulovasine A and B. The isomer 2,8-dichlororugulovasine A represents the major compound within the mixture.

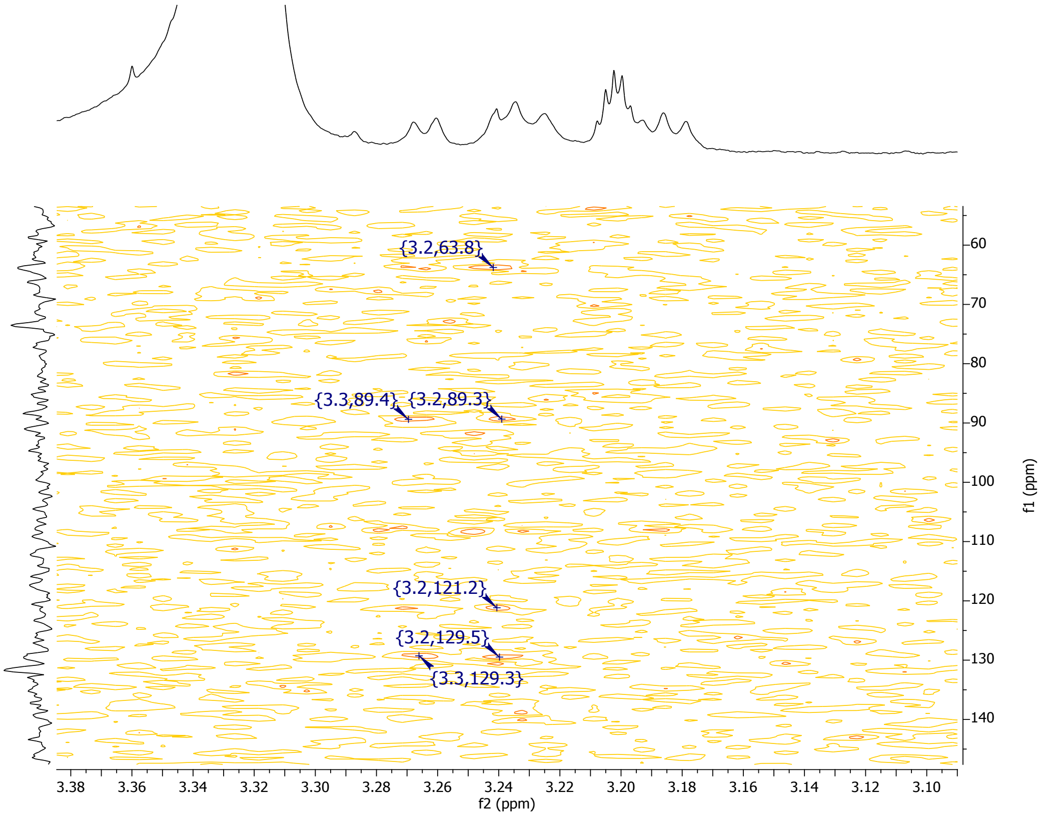

Figure S24. Magnified region of HMBC spectrum from the mixture of 2,8-chlororugulovasine A and B. The isomer 2,8-dichlororugulovasine A represents the major compound within the mixture.

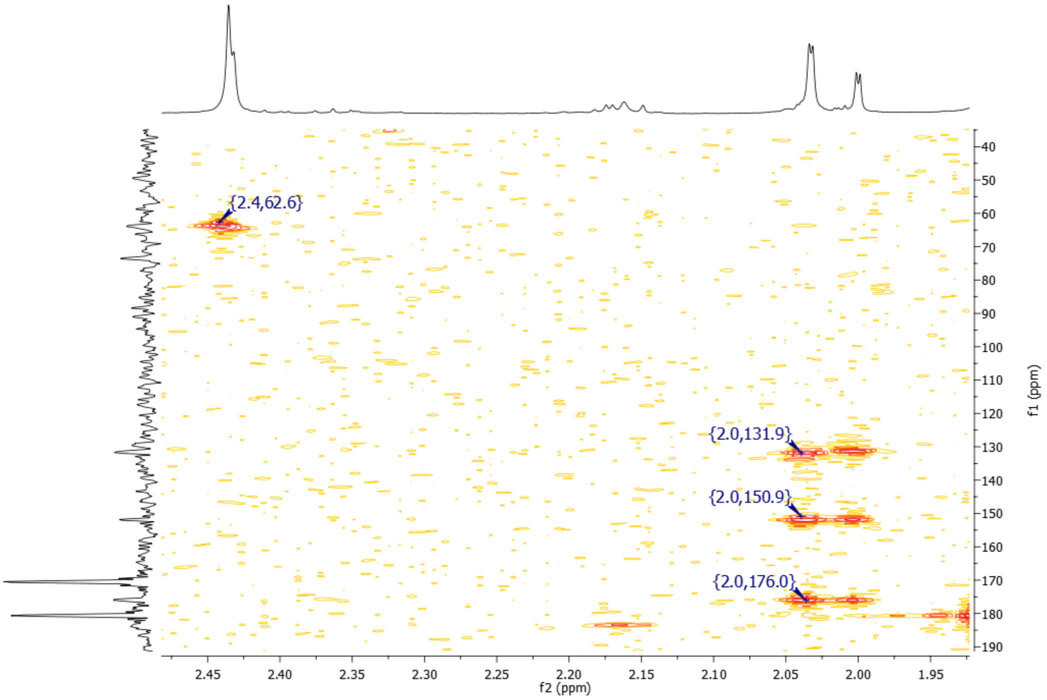

Figure S25. Magnified region of HMBC spectrum from the mixture of 2,8-chlororugulovasine A and B. The isomer 2,8-dichlororugulovasine A represents the major compound within the mixture.

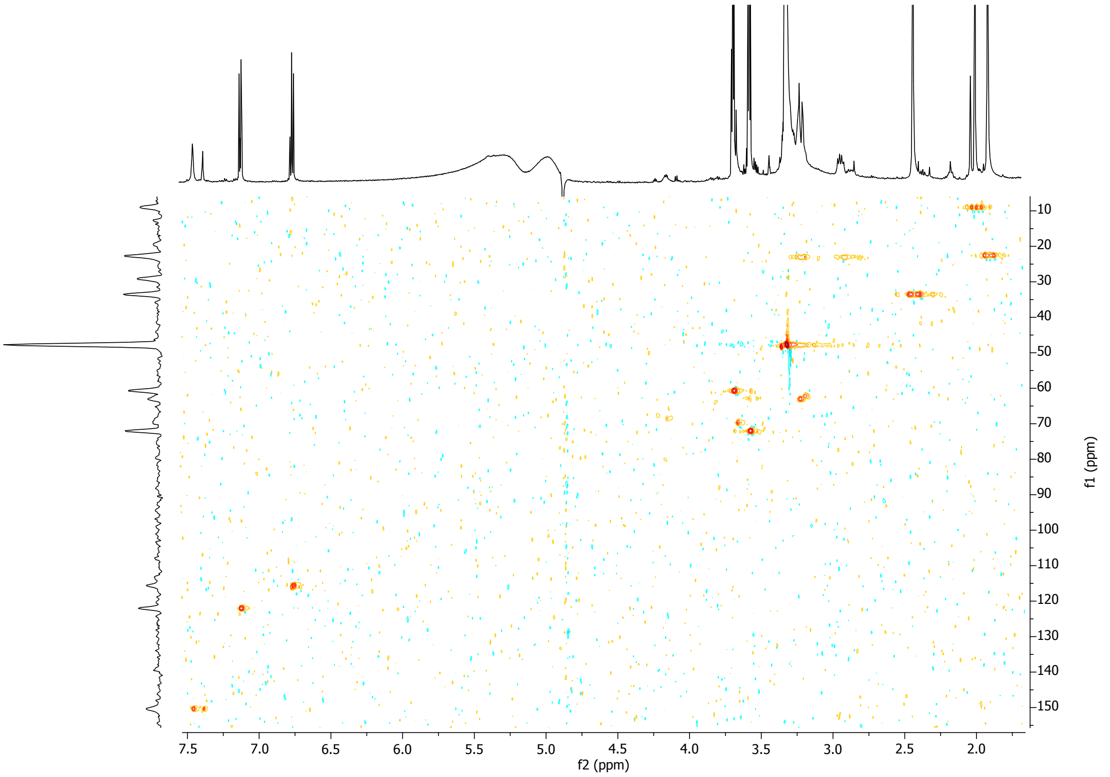

Figure S26. HSQC spectrum from the mixture of 2,8-chlororugulovasine A and B. The isomer 2,8-dichlororugulovasine B represents the major compound within the mixture.

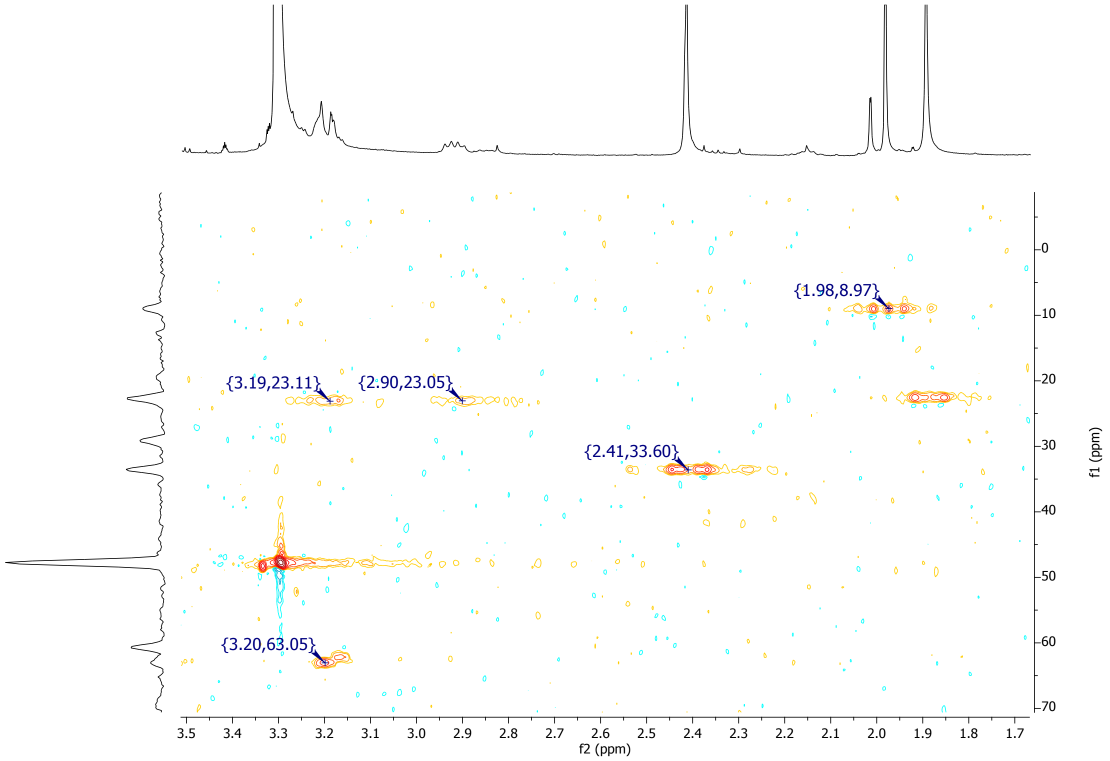

Figure S27. Magnified region of HSQC spectrum from the mixture of 2,8-chlororugulovasine A and B. The isomer 2,8-dichlororugulovasine B represents the major compound within the mixture.

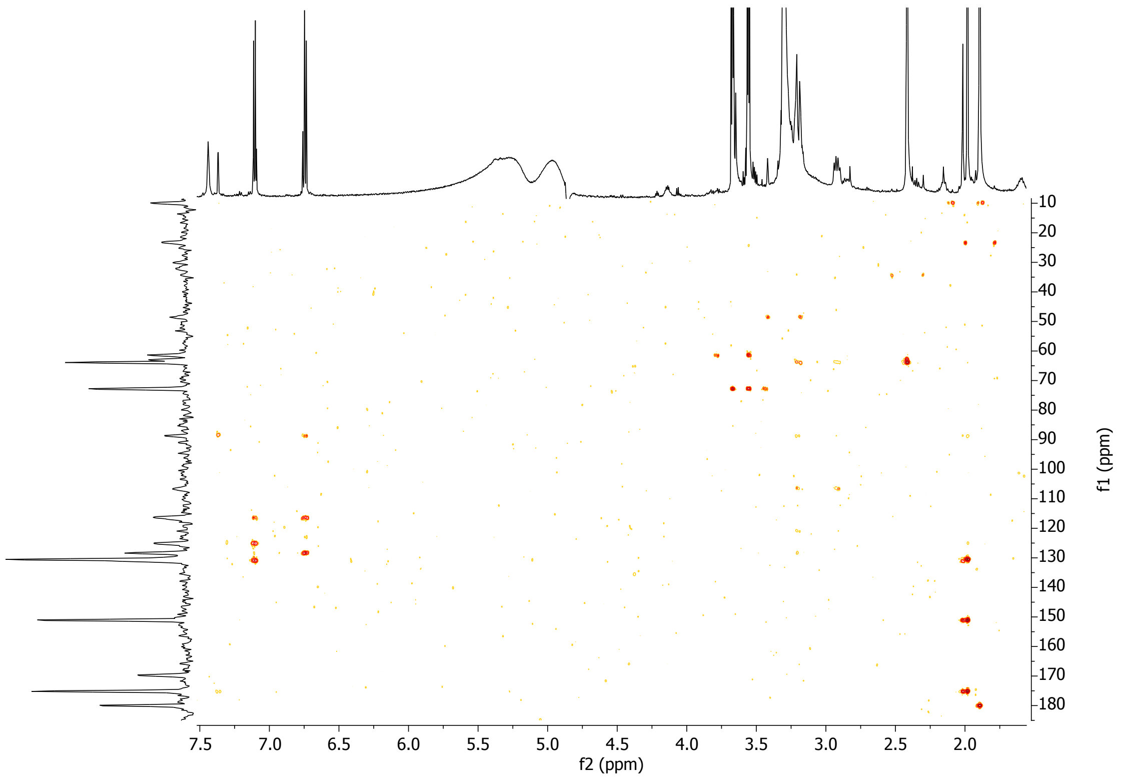

Figure S28. HMBC spectrum from the mixture of 2,8-chlororugulovasine A and B. The isomer 2,8-dichlororugulovasine B represents the major compound within the mixture.

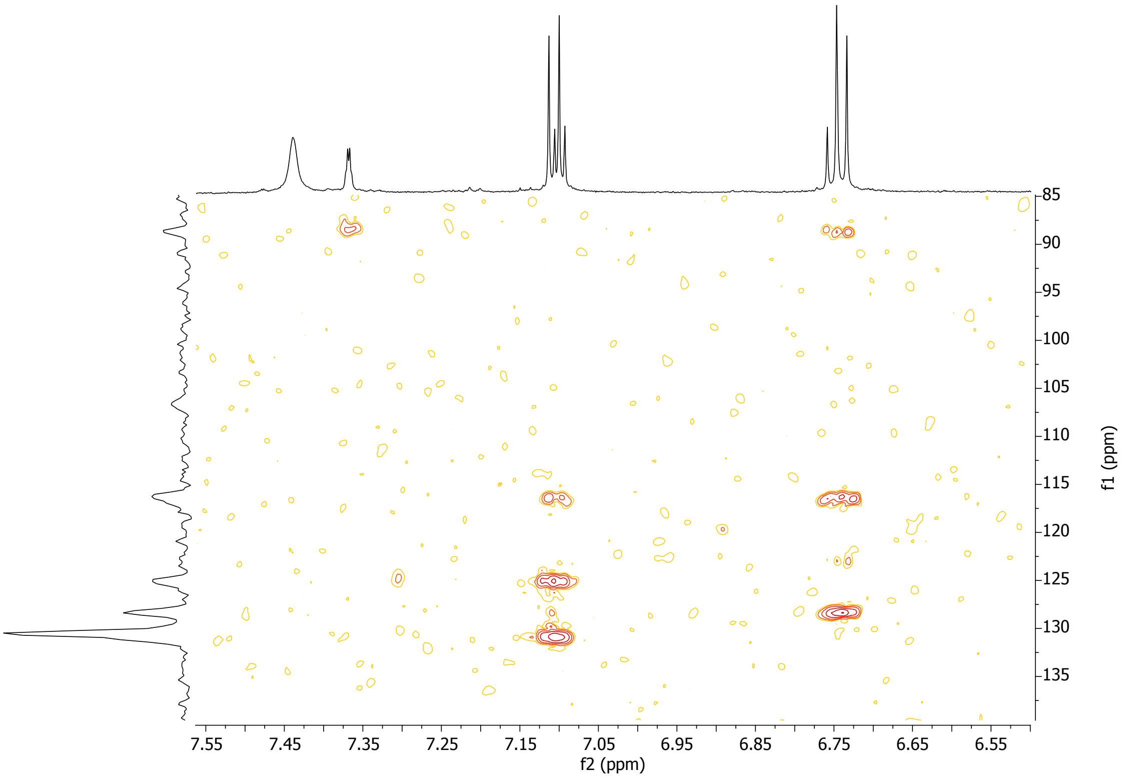

Figure S29. Magnified region of HMBC spectrum from the mixture of 2,8-chlororugulovasine A and B. The isomer 2,8-dichlororugulovasine B represents the major compound within the mixture.

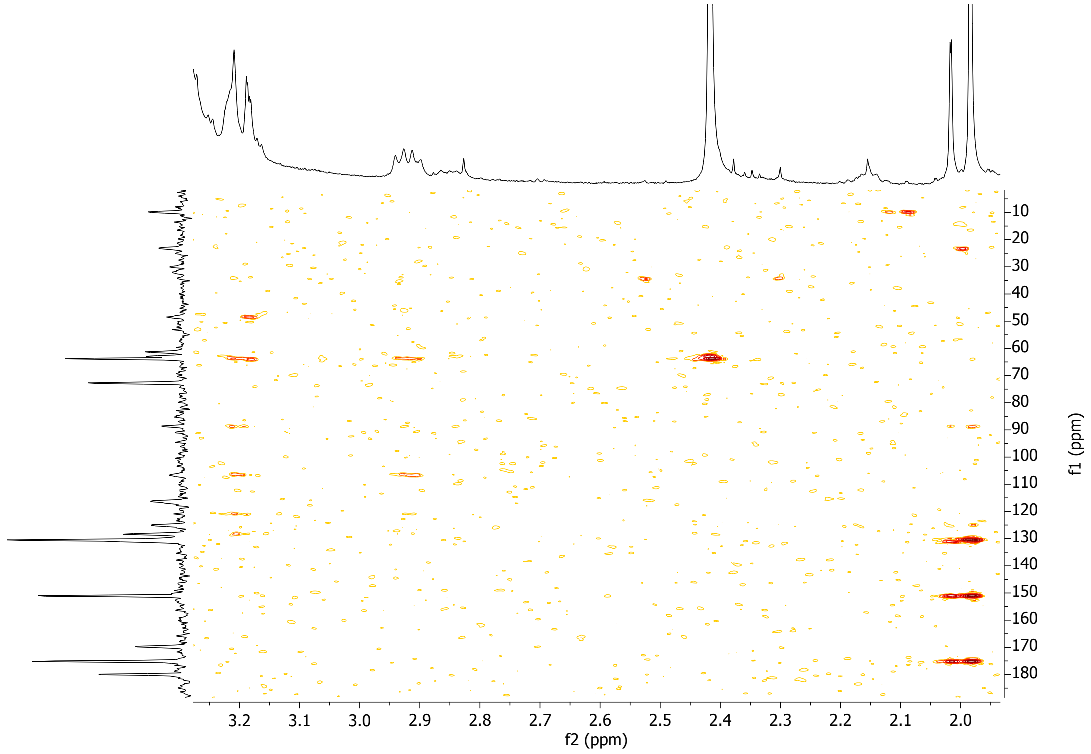

Figure S30. Magnified region of HMBC spectrum from the mixture of 2,8-chlororugulovasine A and B. The isomer 2,8-dichlororugulovasine B represents the major compound within the mixture.

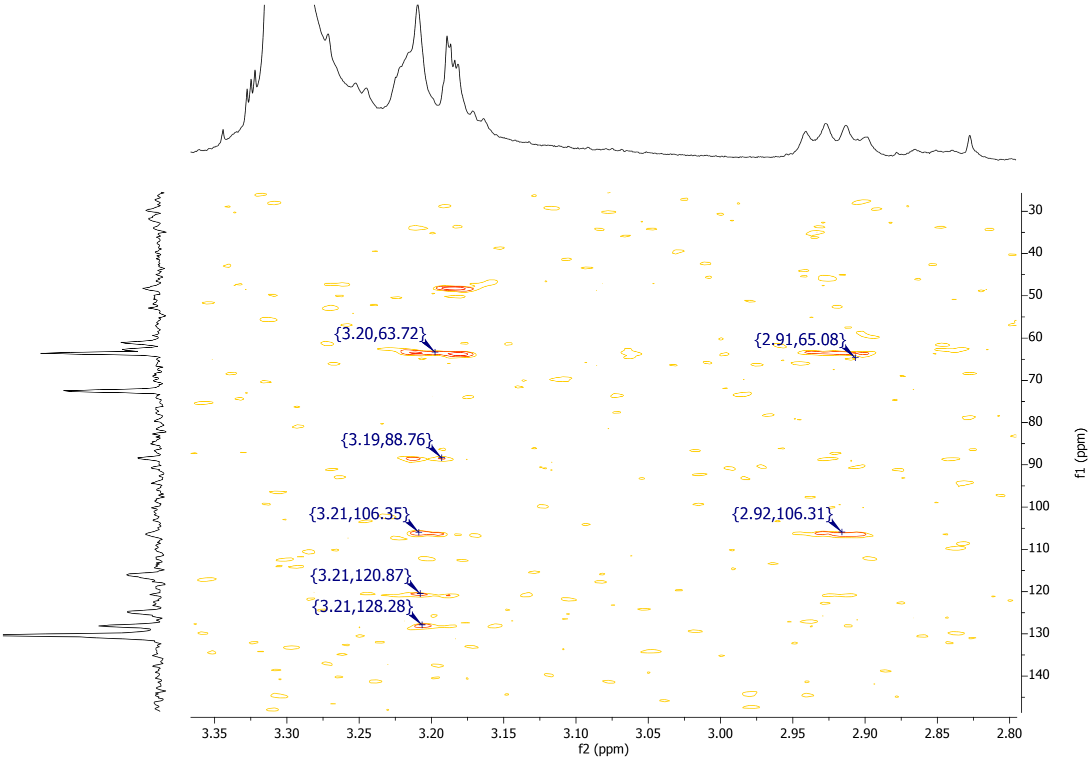

Figure S31. Magnified region of HMBC spectrum from the mixture of 2,8-chlororugulovasine A and B. The isomer 2,8-dichlororugulovasine B represents the major compound within the mixture.

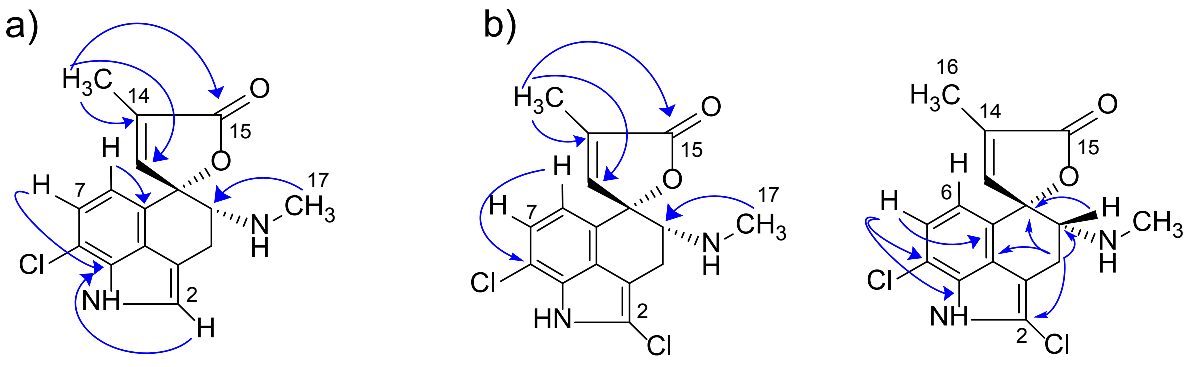

Figure S32. Long-range correlations detected in the HMBC spectra of (**a**) 8-chlororugulovasine A and (**b**) 2,8-dichlororugulovasine A.

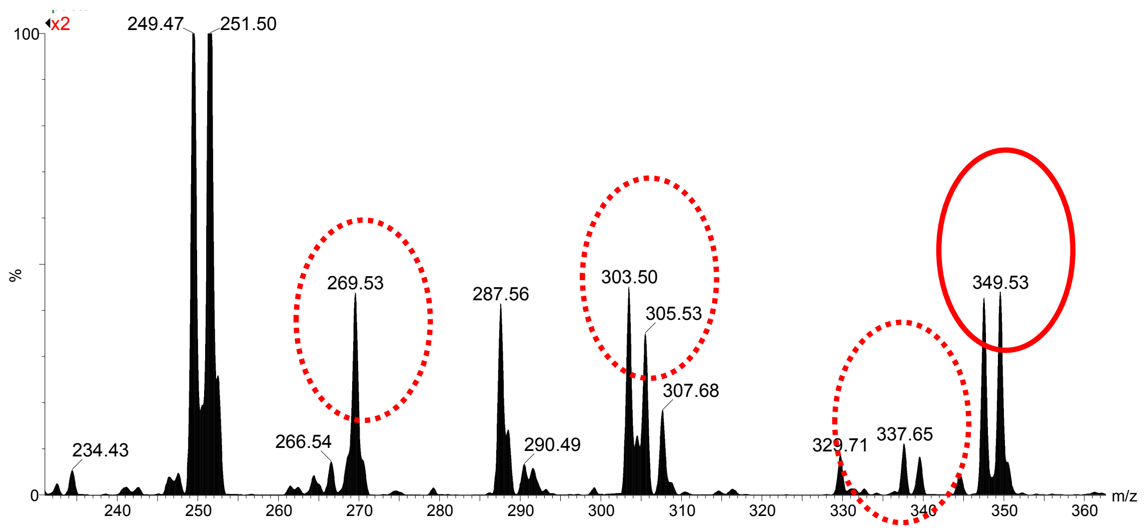

Figure S33. Full scan spectrum of the crude extract obtained from *T. wortmannii* cultivated in PD medium with KBr as additive. Pseudomolecular ions from the co-produced rugulovasines and chlorinated analogues are highlighted in dashed red line while the brominated specie detected is highlighted in continuous red line. Data acquired in ESI+, triple quadrupole-MS.

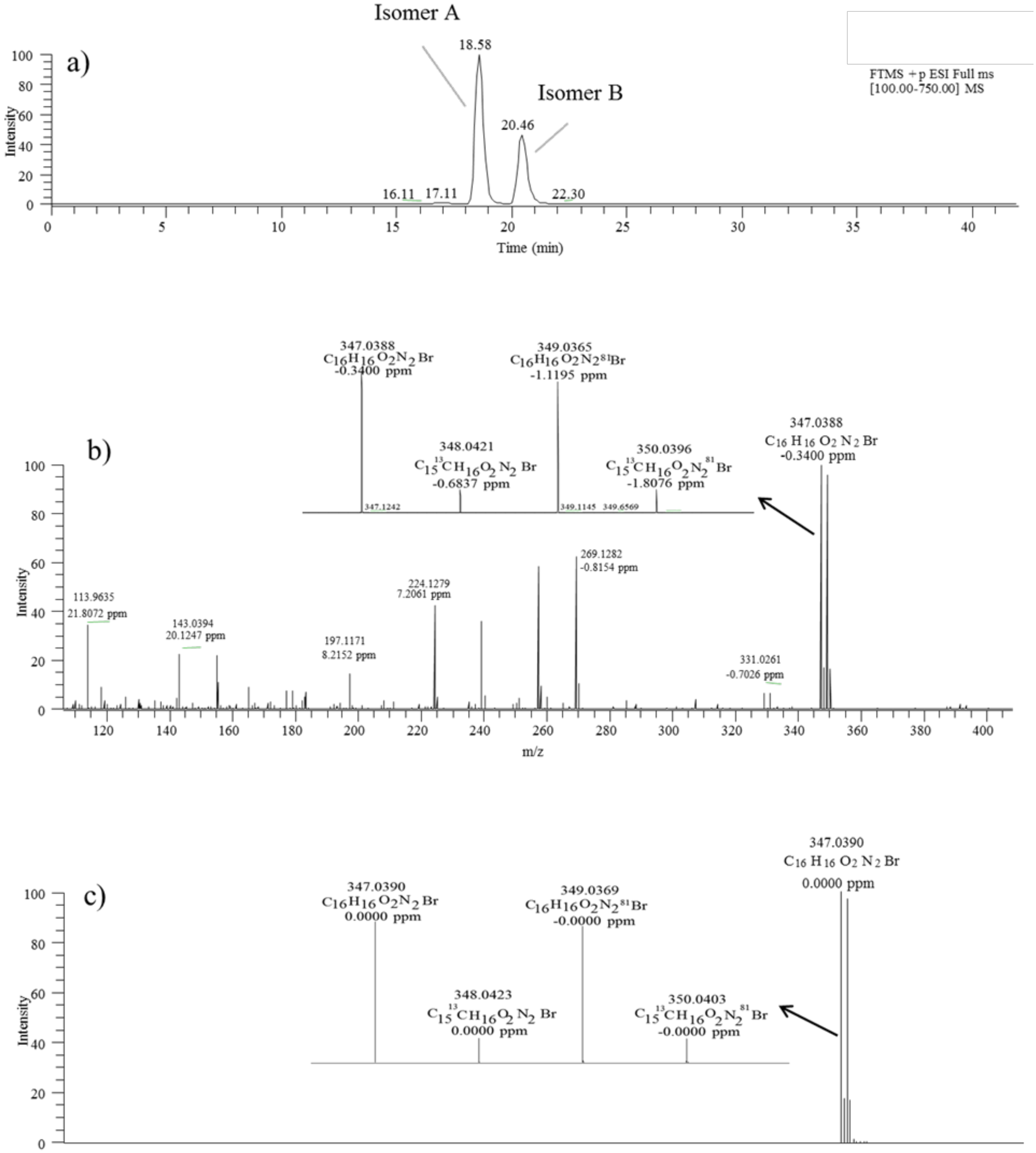

Figure S34. (**a**) EIC (Extracted Ion Chromatogram) of *m/z* 347.0395 (±5 ppm) from the crude extract obtained from *T. wortmannii* cultivated in PD medium with HBr as additive. Two major peaks are observed and indicated as likely isomers A and B from the brominated species; (**b**) HRMS full scan spectrum of the peak highlighted as isomer B in the ECI chromatogram and magnified region corresponding to the accurate mass from the pseudomolecular ion and isotopes; (**c**) Simulated HRMS spectrum, in positive ionization mode from the theoretical pseudomolecular ion obtained for the molecular formula $C_{16}H_{16}O_2N_2Br$ and magnified region of the obtained signal. Data acquired in ESI+, UHPLC-FTMS.

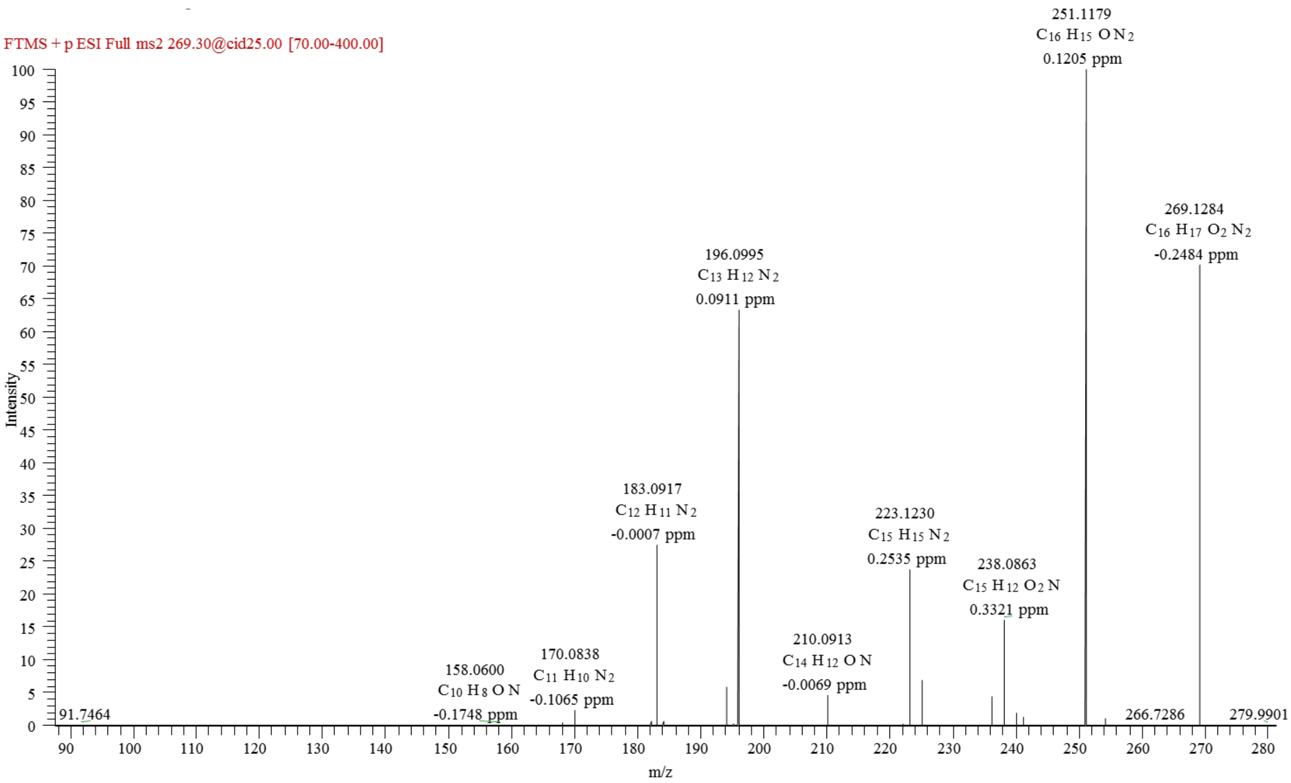

Figure S35. MS/HRMS spectrum of rugulovasine A, ESI+, 25 eV.

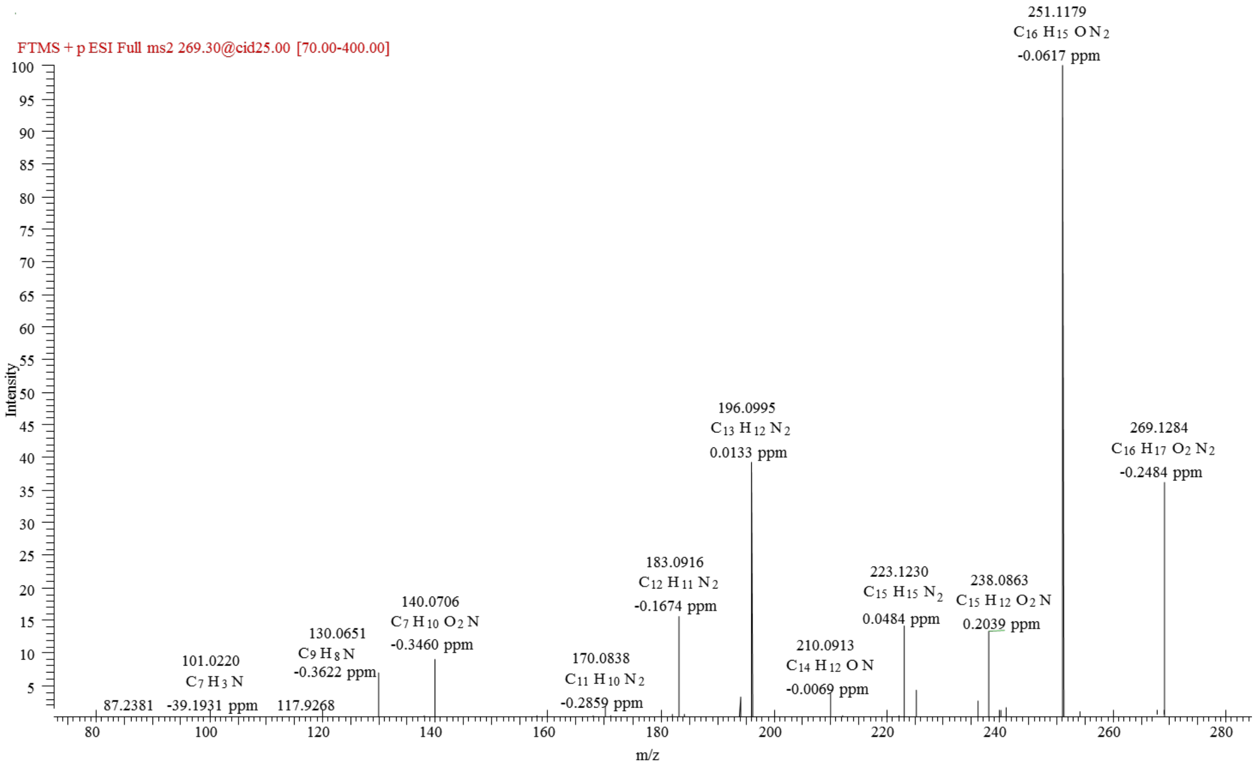

Figure S36. MS/HRMS spectrum of rugulovasine B, ESI+, 25 eV.

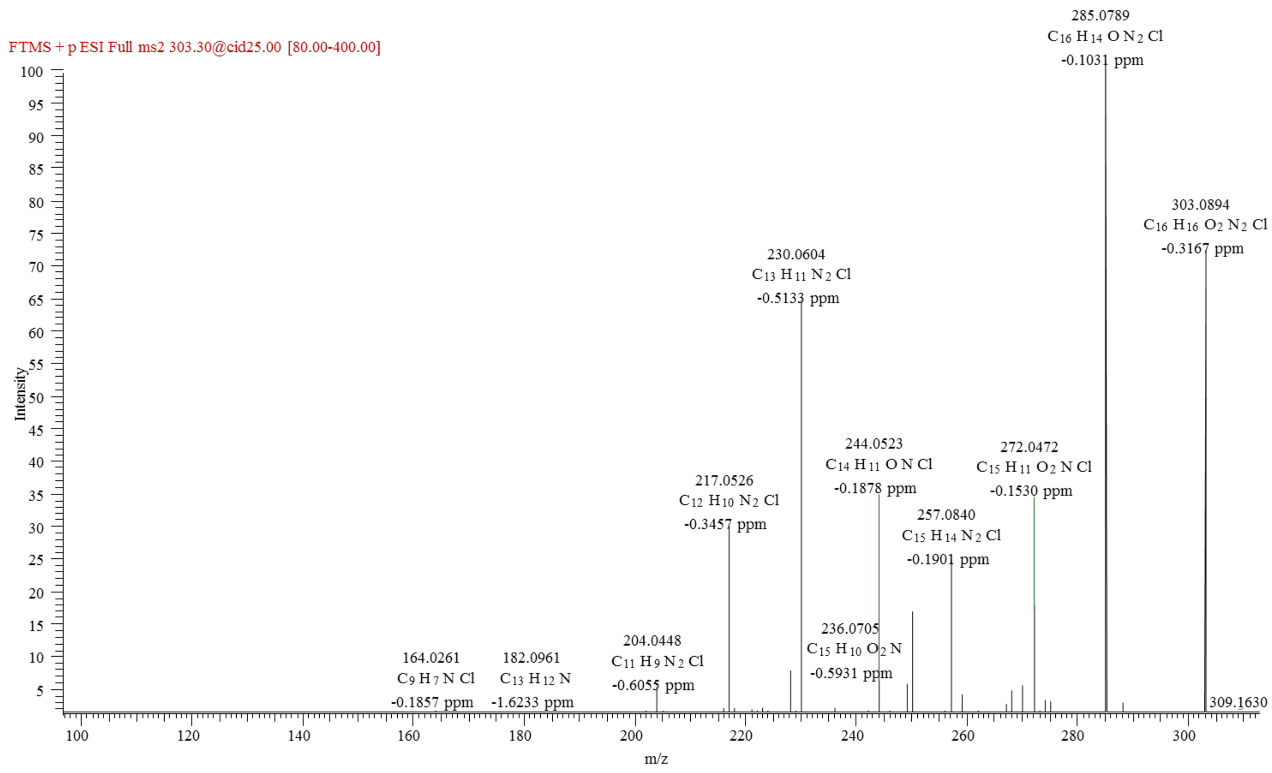

Figure S37. MS/HRMS spectrum of 8-chlororugulovasine A, ESI+, 25 eV.

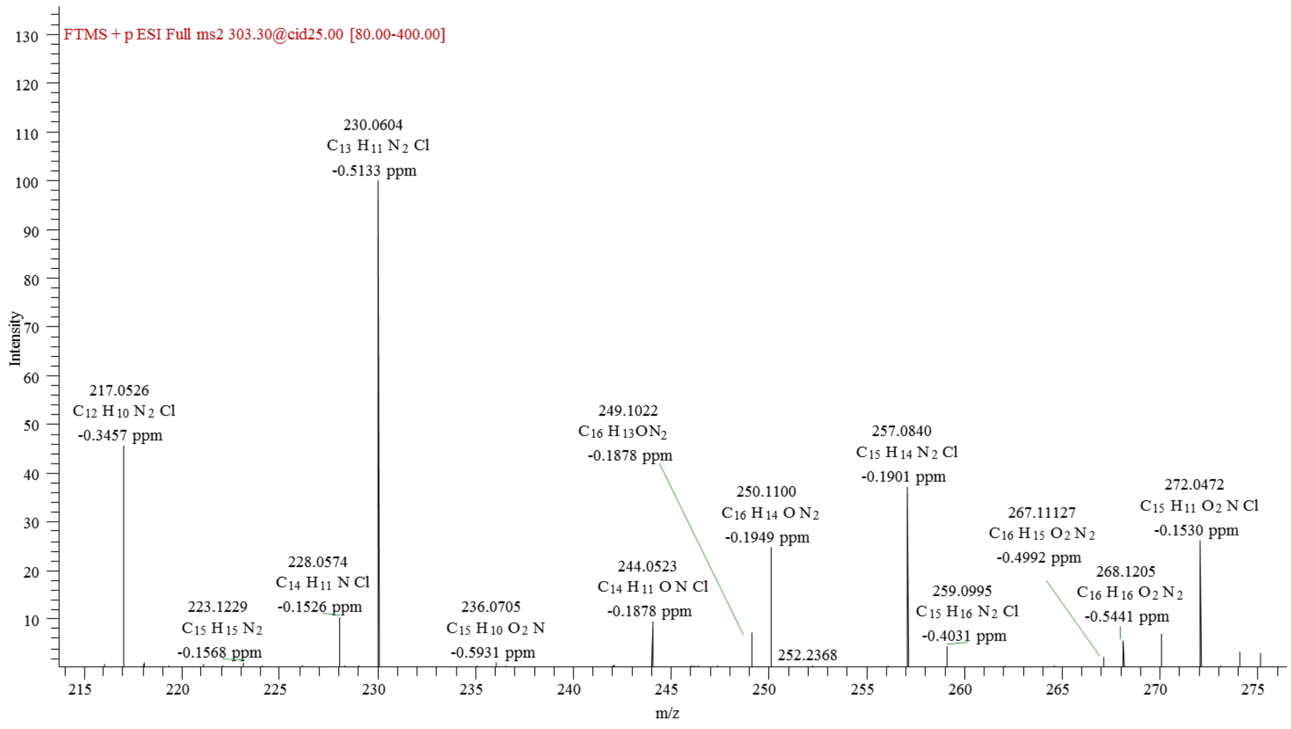

Figure S38. Magnified region from MS/HRMS spectrum of 8-chlororugulovasine A, ESI+, 25 eV.

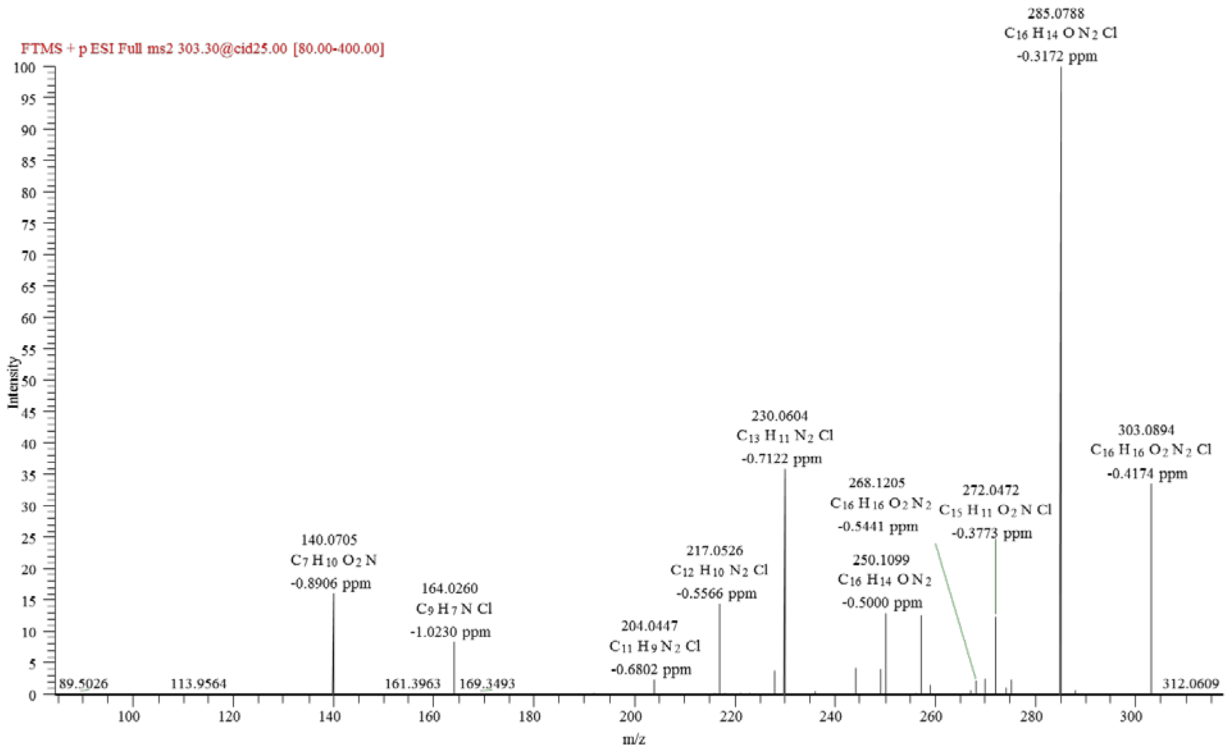

Figure S39. MS/HRMS spectrum of 8-chlororugulovasine B, ESI+, 25 eV

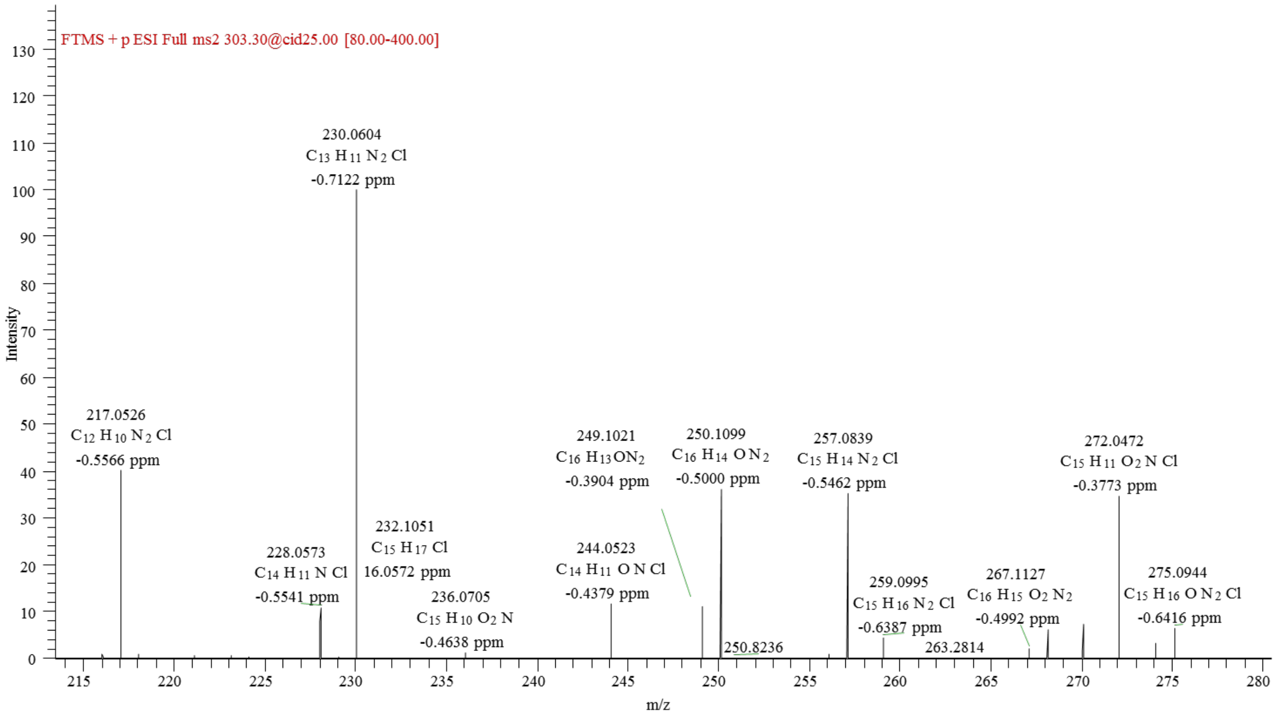

Figure S40. Magnified region from MS/HRMS spectrum of 8-chlororugulovasine B, ESI+, 25 eV.

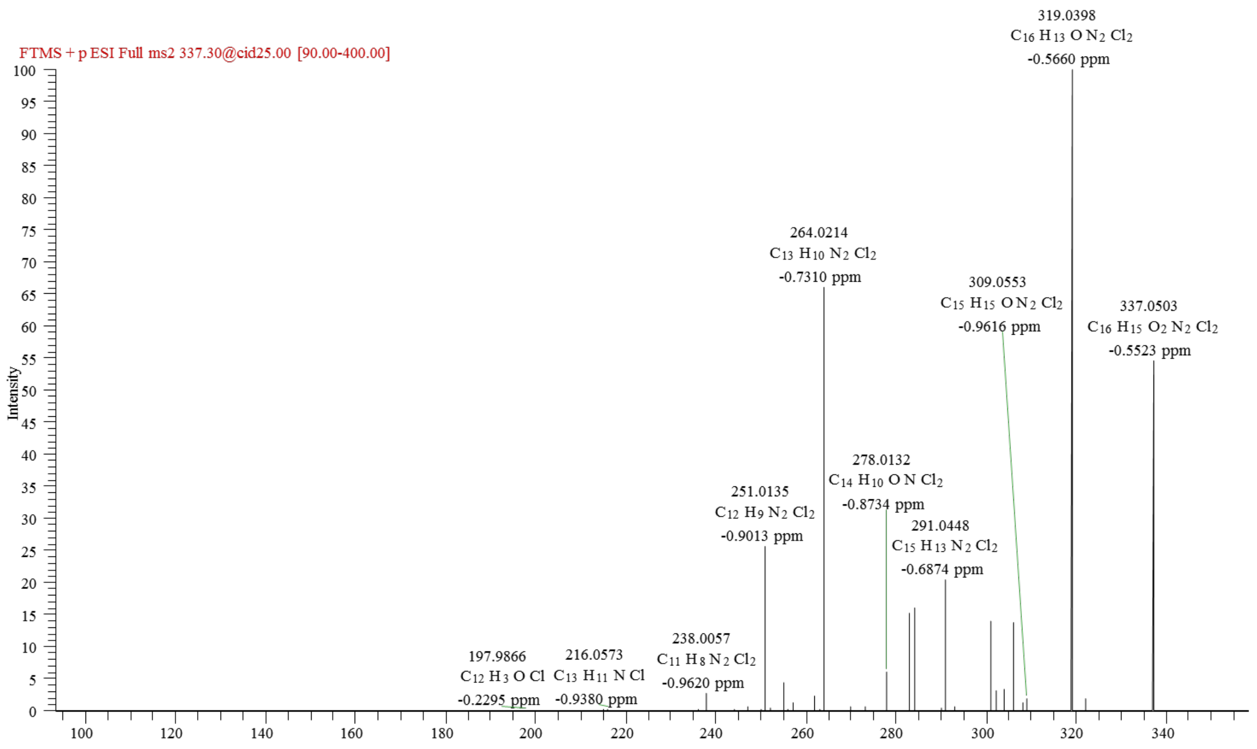

Figure S41. MS/HRMS spectrum of the new 2,8-dichlororugulovasine A, ESI+, 25 eV.

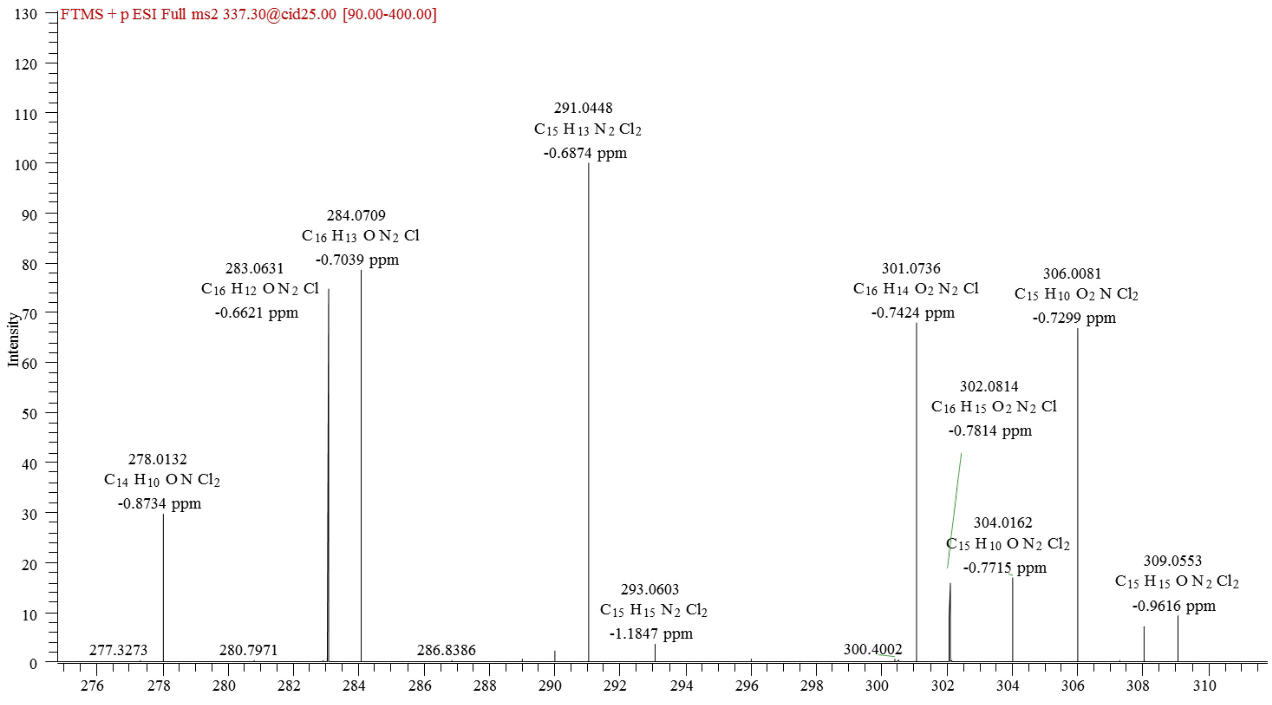

Figure S42. Magnified region from MS/HRMS spectrum of new 2,8-dichlororugulovasine A, ESI+, 25 eV.

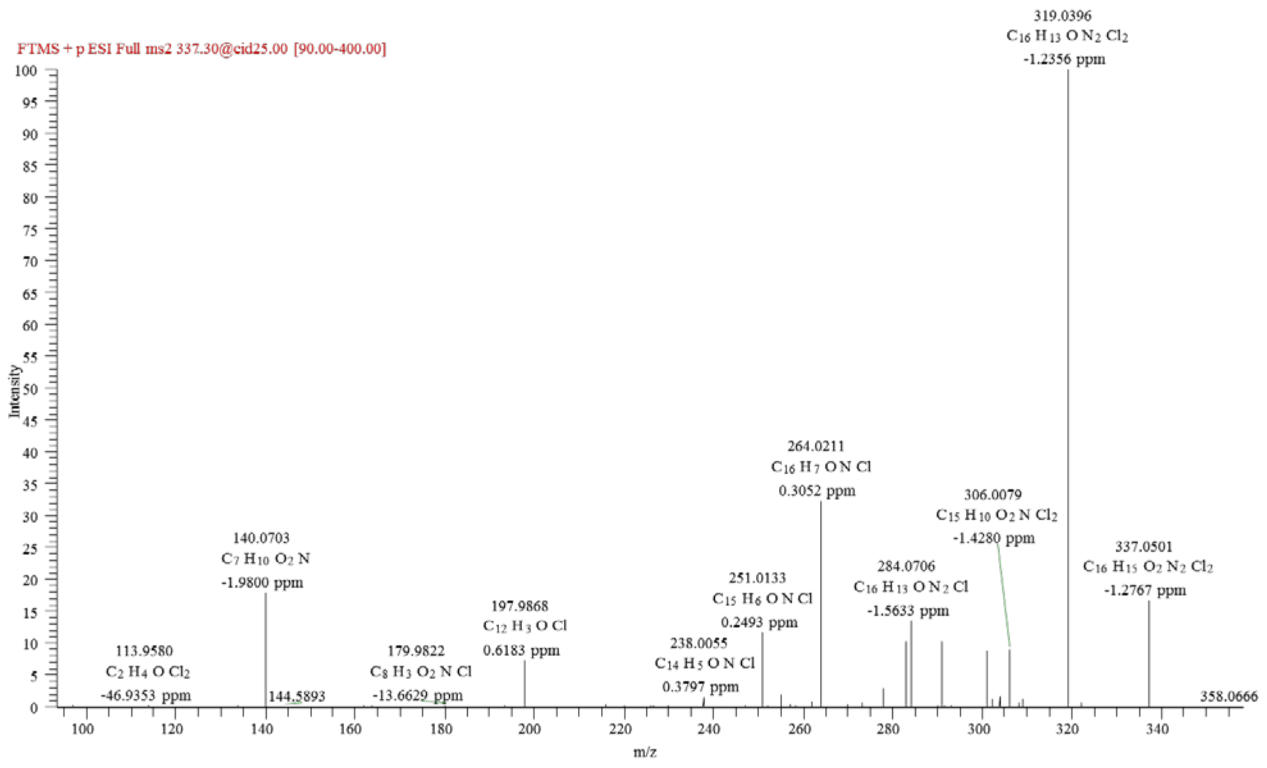

Figure S43. MS/HRMS spectrum of the new 2,8-dichlororugulovasine B, ESI+, 25 eV.

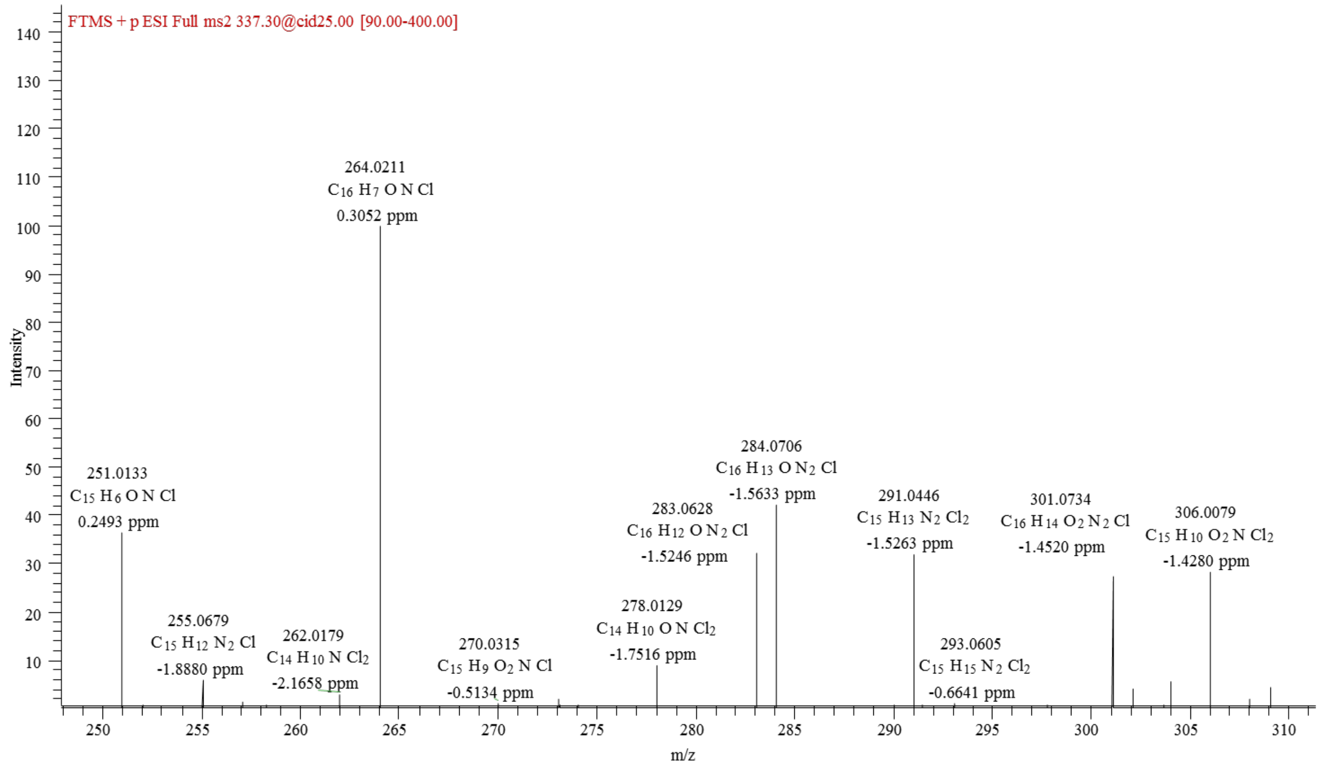

Figure S44. Magnified region from MS/HRMS spectrum of new 2,8-dichlororugulovasine B, ESI+, 25 eV.

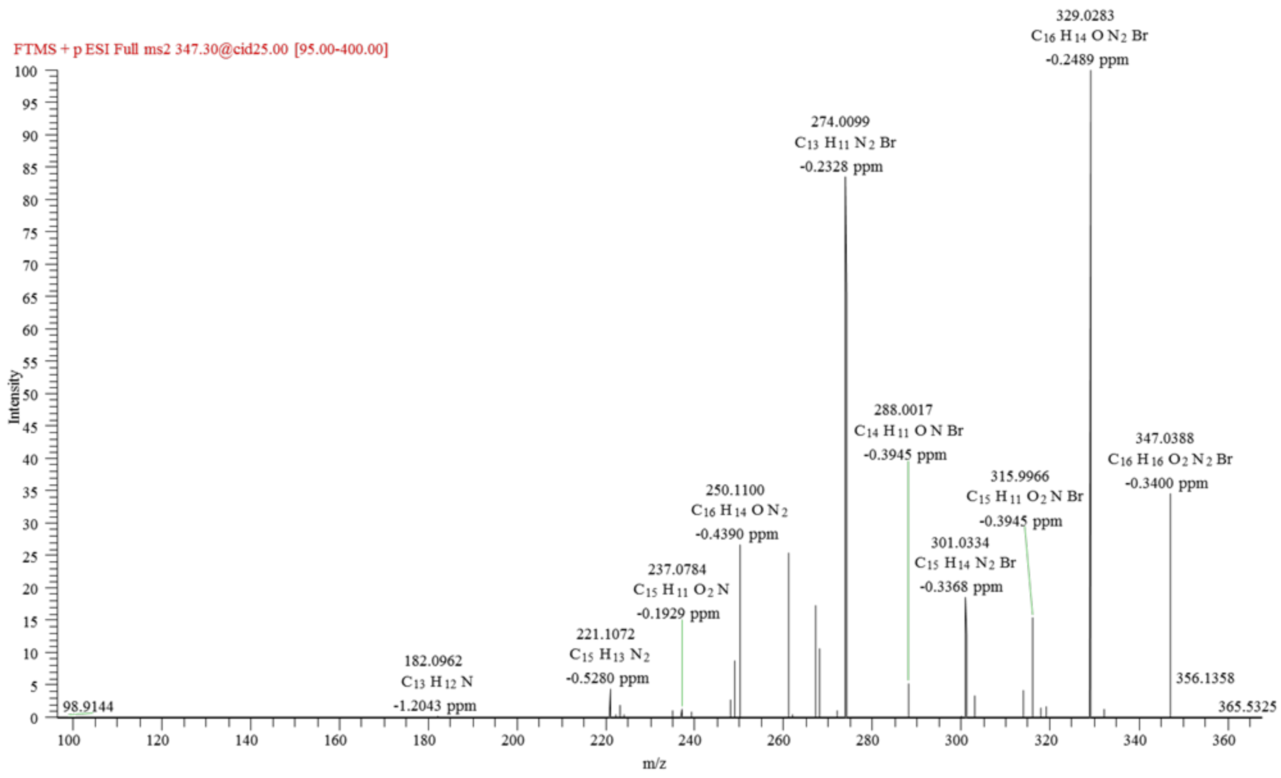

Figure S45. MS/HRMS spectrum of the new 8-bromorugulovasine A, ESI +, 25 eV

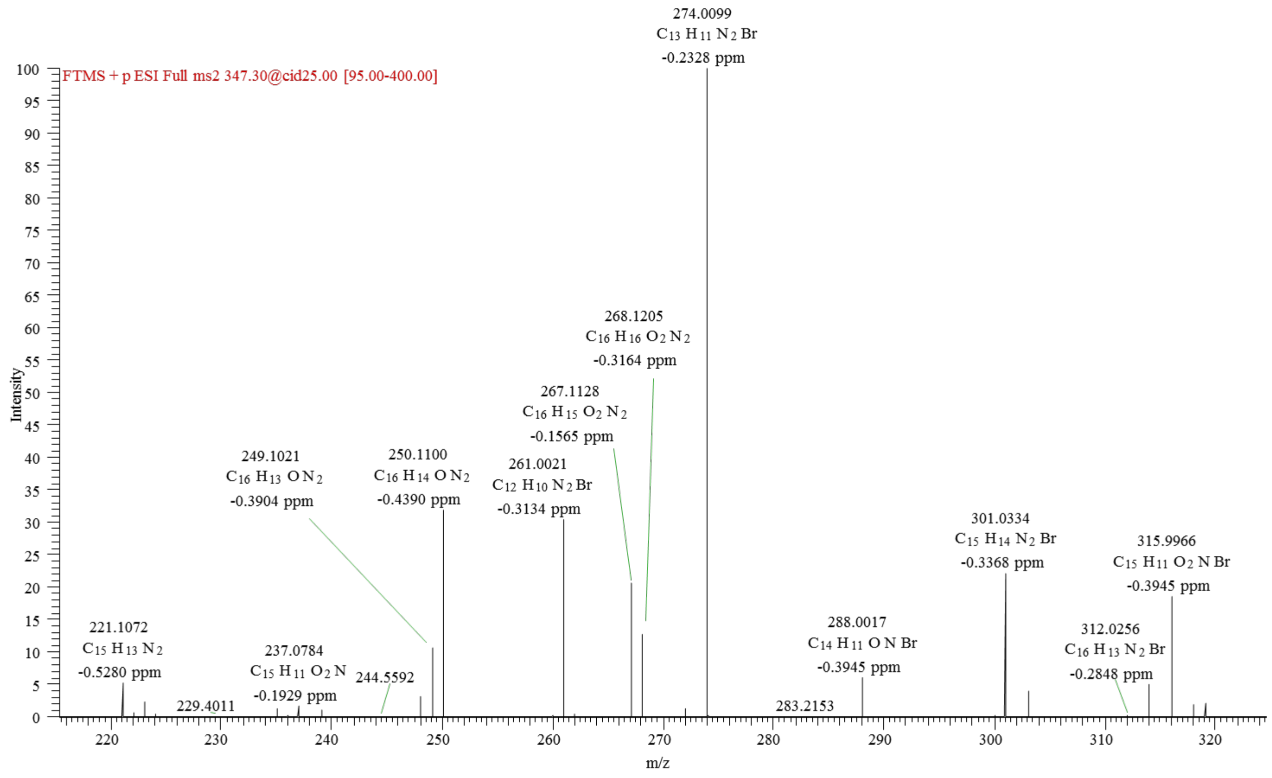

Figure S46. Magnified region from MS/HRMS spectrum of 8-bromorugulovasine A, ESI+, 25 eV.

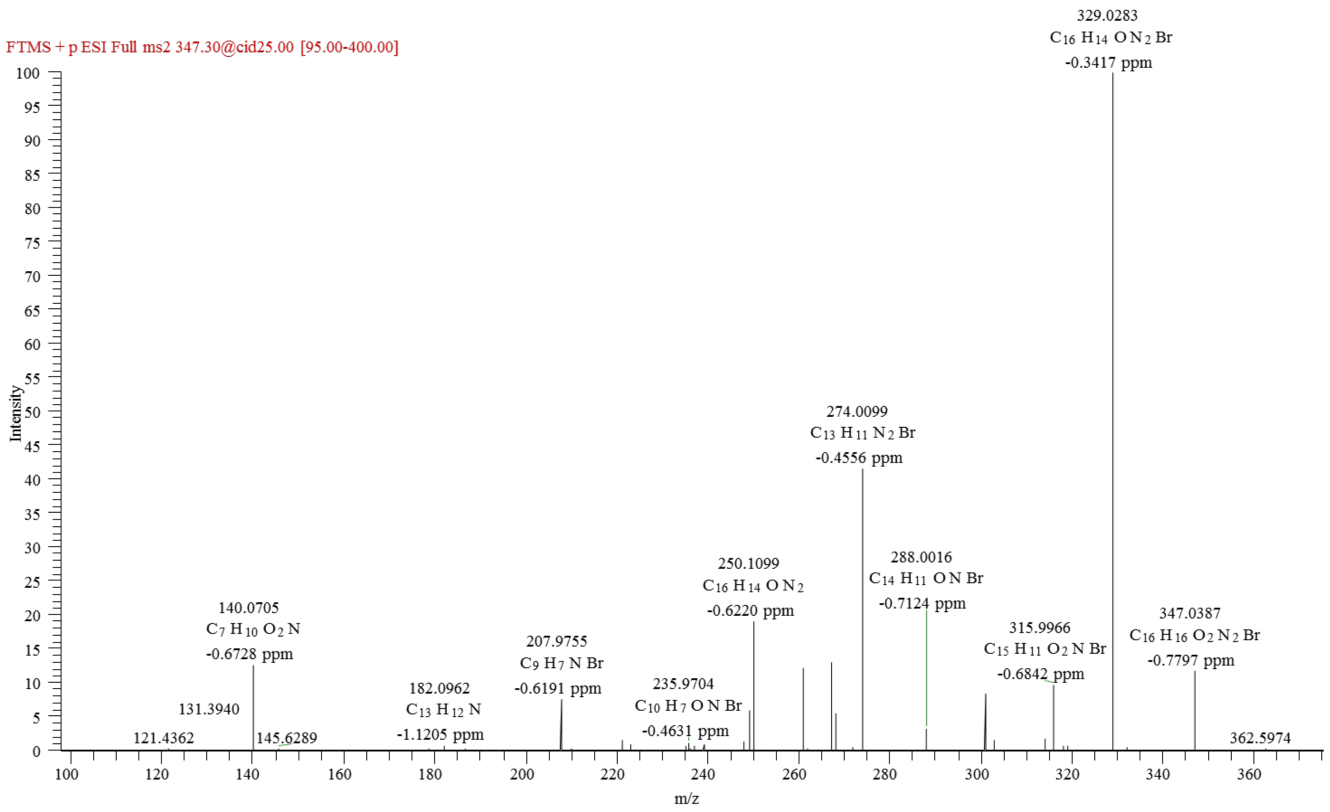

Figure S47. MS/HRMS spectrum of the new 8-bromorugulovasine B, ESI +, 25 eV

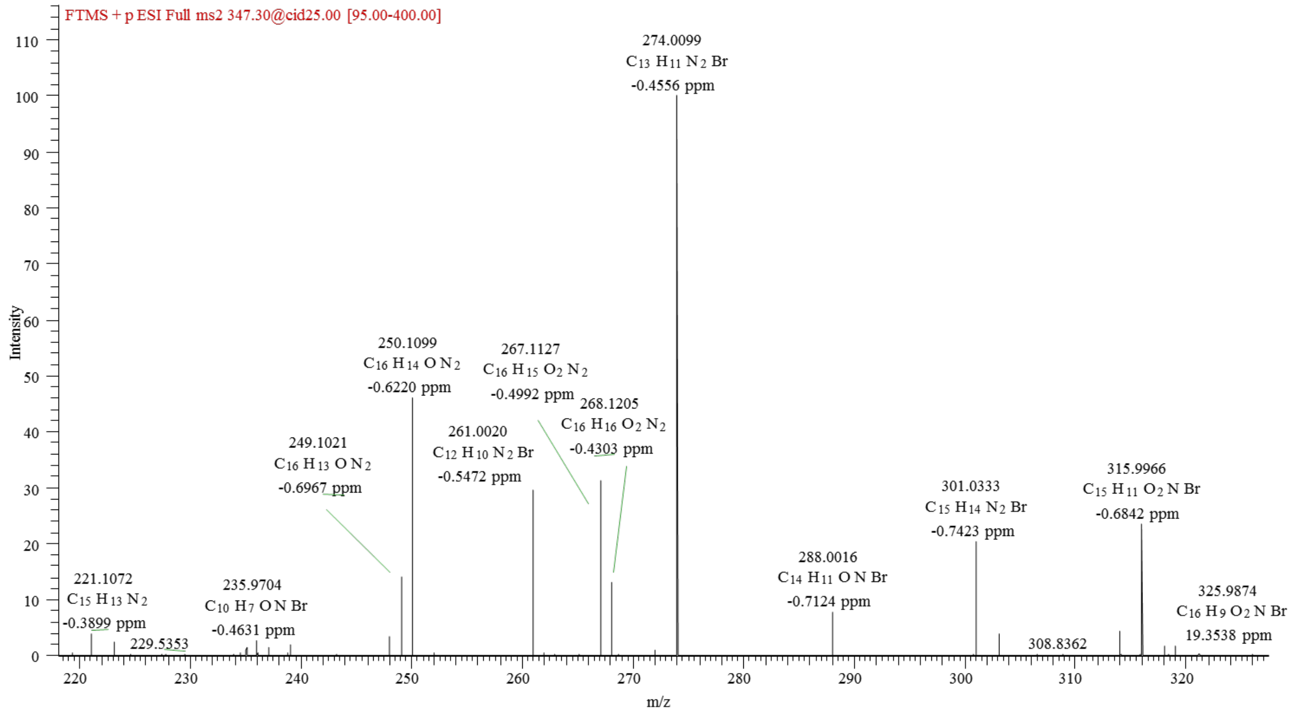

Figure S48. Magnified region MS/HRMS spectrum of the new 8-bromorugulovasine B, ESI+, 25 eV.

1 and **2**: R_1 = H; R_2 = H **5** and **6**: R_1 = Cl; R_2 = Cl
3 and **4**: R_1 = Cl; R_2 = H **7** and **8**: R_1 = H; R_2 = Br

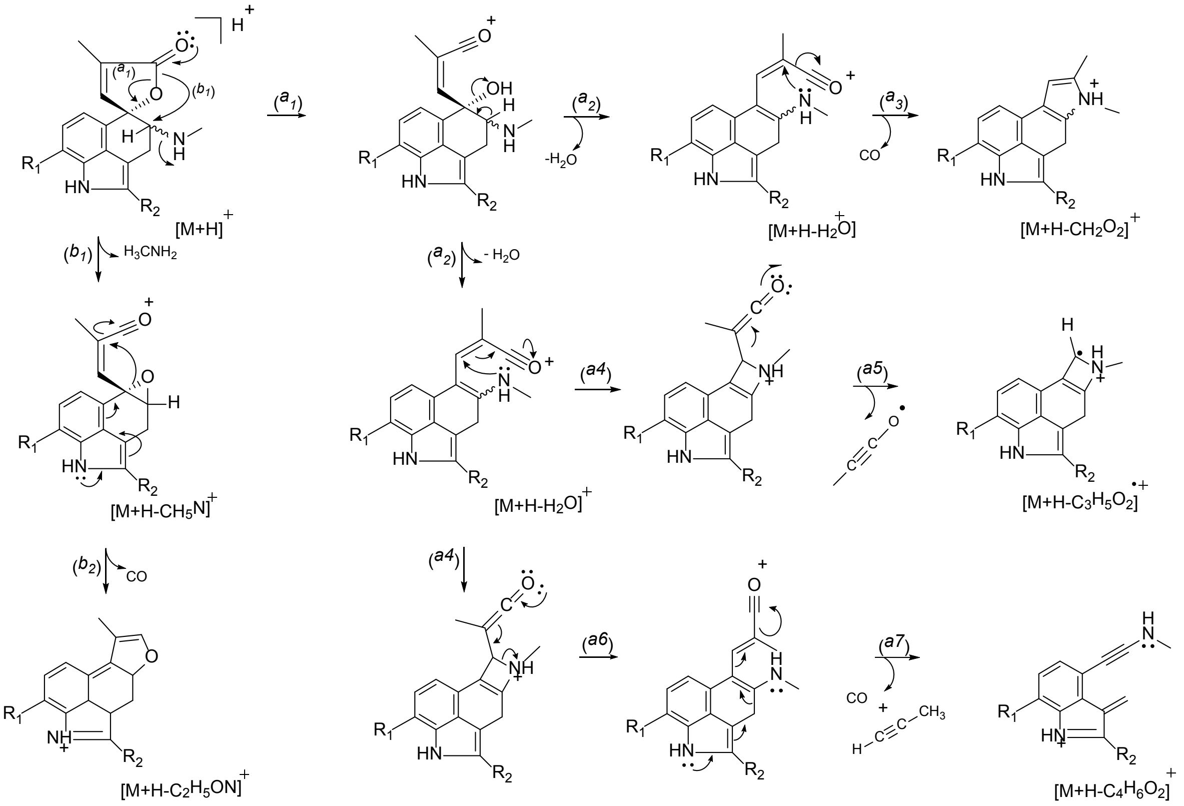

Figures S49. Fragmentation mechanisms proposed to explain the product ions described in Table S2, regarding the detected species in the MS/HRMS spectra of compounds **1**–**8**. The observed fragment ions assist the rugulovasine backbone for the studied compounds. See Table S2.

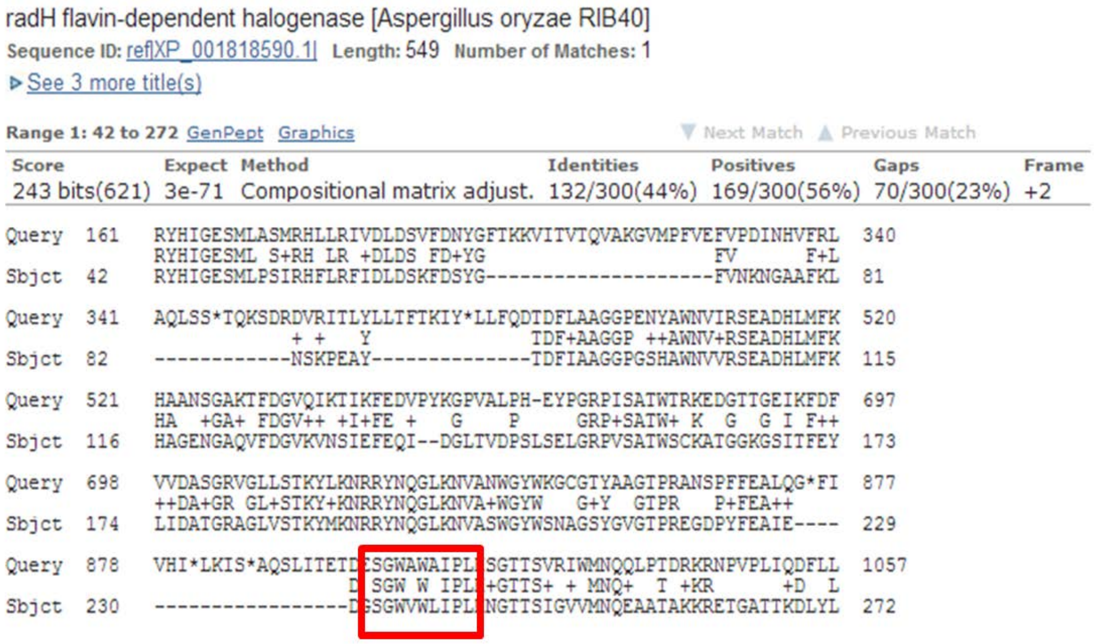

radH flavin-dependent halogenase [Aspergillus oryzae RIB40]
Sequence ID: ref|XP_001818590.1| Length: 549 Number of Matches: 1
▷ See 3 more title(s)

Range 1: 42 to 272 GenPept Graphics ▼ Next Match ▲ Previous Match

Score	Expect	Method	Identities	Positives	Gaps	Frame
243 bits(621)	3e-71	Compositional matrix adjust.	132/300(44%)	169/300(56%)	70/300(23%)	+2

```
Query  161   RYHIGESMLASMRHLLRIVDLDSVFDNYGFTKKVITVTQVAKGVMPFVEFVPDINHVFRL  340
             RYHIGESML S+RH LR +DLDS FD+YG                    FV       F+L
Sbjct  42    RYHIGESMLPSIRHFLRFIDLDSKFDSYG--------------------FVNKNGAAFKL  81

Query  341   AQLSS*TQKSDRDVRITLYLLTFTKIY*LLFQDTDFLAAGGPENYAWNVIRSEADHLMFK  520
                          + +   Y              TDF+AAGGP ++AWNV+RSEADHLMFK
Sbjct  82    ------------NSKPEAY--------------TDFIAAGGPGSHAWNVVRSEADHLMFK  115

Query  521   HAANSGAKTFDGVQIKTIKFEDVPYKGPVALPH-EYPGRPISATWTRKEDGTTGEIKFDF  697
             HA  +GA+ FDGV++ +I+FE +   G    P      GRP+SATW+ K  G  G I F++
Sbjct  116   HAGENGAQVFDGVKVNSIEFEQI--DGLTVDPSLSELGRPVSATWSCKATGGKGSITFEY  173

Query  698   VVDASGRVGLLSTKYLKNRRYNQGLKNVANWGYWKGCGTYAAGTPRANSPFFEALQG*FI  877
             ++DA+GR GL+STKY+KNRRYNQGLKNVA+WGYW   G+Y  GTPR   P+FEA++
Sbjct  174   LIDATGRAGLVSTKYMKNRRYNQGLKNVASWGYWSNAGSYGVGTPREGDPYFEAIE----  229

Query  878   VHI*LKIS*AQSLITETD[illegible]SGWAWAIPL[illegible]SGTTSVRIWMNQQLPTDRKRNPVPLIQDFLL  1057
                               D SGW W IPL[illegible]+GTTS+ + MNQ+  T +KR      +D  L
Sbjct  230   ----------------D[illegible]SGWVWLIPL[illegible]NGTTSIGVVMNQEAATAKKRETGATTKDLYL  272
```

Figure S50. Comparison for the gene fragment isolated from *T. wortmannii*. Amino-acid conserved region for FADH dependent halogenases highlighted in red line.

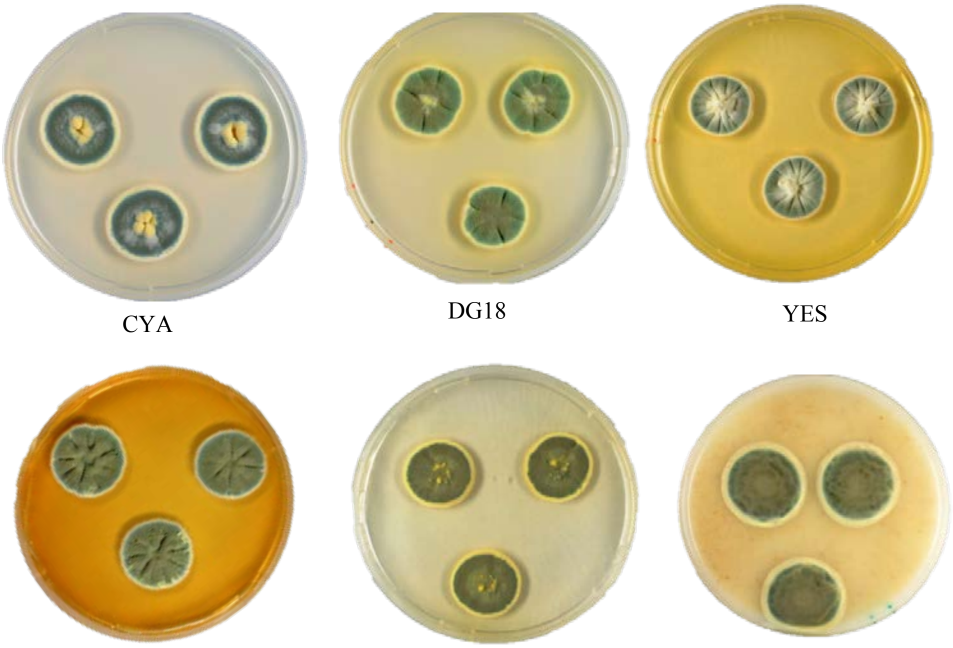

Figure S51. *Talaromyces wortmanii* cultivated for seven days, 25 °C, in the dark, on six different media: Czapek yeast autolysate agar (CYA), dichloran 18% glycerol agar (DG18), yeast extract sucrose agar (YES), malt extract agar (Oxoid) (MEAox), potato dextrose agar (PDA), and oatmeal agar (OA).

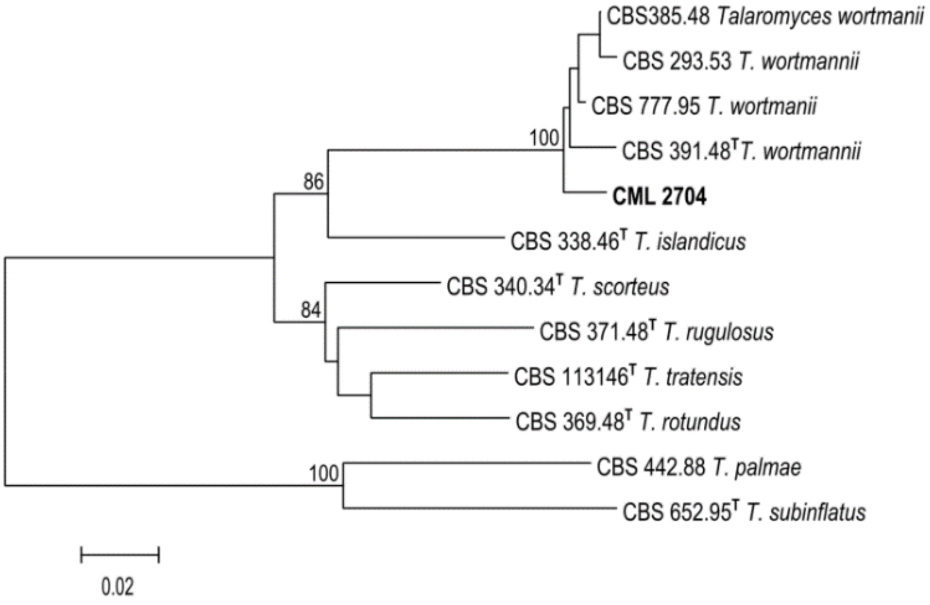

Figure S52. Maximum Likelihood phylogenetic tree of partial RPB2 (RNA polymerase II second-largest subunit) sequences of strain CML 2704 and reference strains of *Talaromyces wortmanii* and related species. Bootstrap values (1000 pseudo replicates) equal or higher than 70% are showed above nodes. *Talaromyces palmae* (CBS 442.88) and *Talaromyces subinflatus* (CBS 652.95) were used as outgroup. ^{T} identifies type specimens.

Table S1. ^{1}H and HMBC data (600 MHz, methanol-d_4) of 8-chlororugulovasine A and B.

Position	8-Chlororugulovasine A			8-Chlororugulovasine B
	δ_H (*J* in Hz)	δ_C *	HMBC	δ_H (*J* in Hz)
1	8.50 (s)	-	-	8.45 (s)
2	7.20 (br s)	122.9	C-9	7.17 (br l)
3	-	-	-	-
4	-	-	-	-
5	-	129.1	-	-
6	6.77 (d, 7.7)	116.7	C-5	6.72 (d, 7.7)
7	7.13 (d, 7.7)	123.3	C-9	7.11 (d, 7.7)
8	-	**	-	-
9	-	132.7	-	-
10	***	-	-	***
11	***	63.7	-	***
12	-	88.5	-	-
13	7.40 (br q, 1.4)	150.2	C-12	7.44 (br s)
14	-	131.4	-	-
15	-	175.3	-	-
16	2.02 (d, 1.3)	10.4	C-13, C-14, C-15	1.98 (d, 1.5)
17	2.53 (br s)	34.3	C-11	2.49 (s)

* Data obtained by 13C projection in HSQC and HMBC experiments; ** Signal not detected; *** Signal not detected in CD3OD due to signal overlapping from solvent.

Table S2. Summary of accurate masses from compounds **1**–**8** and their product ions detected in the MS/HRMS spectra, supporting the species illustrated in Figure S49.

Compound	$[M + H]^+$	$[M + H - H_2O]^+$	$[M + H - CH_2O_2]^+$	$[M + H - CH_5N]^+$	$[M + H - C_2H_5ON]^+$	$[M + H - C_3H_5O_2]^+$	$[M + H - C_4H_6O_2]^+$
(1) rugulovasine A	269.1284	251.1179	223.1220	238.0863	210.0913	196.0995	183.0917
(2) rugulovasine B	269.1284	251.1179	223.1230	238.0863	210.0913	196.0995	183.0916
(3) 8-chlororugulovasine A	303.0894	285.0789	257.0840	272.0472	244.0523	230.0604	217.0526
(4) 8-chlororugulovasine B	303.0894	285.0788	257.0839	272.0472	244.0523	230.0604	217.0526
(5) 2,8-dichlororugulovasine A	337.0503	319.0398	291.0448	306.0081	278.0132	264.0214	251.0135
(6) 2,8-dichlororugulovasine B	337.0501	319.0396	291.0446	306.0081	278.0129	264.0211	251.0133
(7) 2-bromorugulovasine A	347.0388	329.0283	301.0334	315.9966	288.0017	274.0099	261.0021
(8) 2-bromorugulovasine B	347.0387	329.0283	301.0333	315.9966	288.0016	274.0099	261.0021
